# Trends in the burden of most common obesity‐related cancers in 16 Southern Africa development community countries, 1990–2019. Findings from the global burden of disease study

**DOI:** 10.1002/osp4.715

**Published:** 2023-11-21

**Authors:** Philimon Gona, Clara Gona, Suha Ballout, Chabila Mapoma, Sowmya Rao, Ali Mokdad, (GBD 2019 SADC BMI)

**Affiliations:** ^1^ University of Massachusetts Boston Boston Massachusetts USA; ^2^ MGH Institute for Health Professions School of Nursing Boston Massachusetts USA; ^3^ University of Zambia Lusaka Zambia; ^4^ Boston University School of Public Health Boston Massachusetts USA; ^5^ University of Washington Medical School Seattle Washington USA

**Keywords:** annual rate of change, global burden of disease, obesity‐related cancers, prevalence and mortality rates, Southern Africa development community

## Abstract

**Background:**

Obesity‐related cancers in the 16 Southern African Development Community (SADC) countries is quite prominent. The changes and time trends of the burden of obesity‐related cancers in developing countries like SADC remain largely unknown. A descriptive epidemiological analysis was conducted to assess the burden of obesity‐related cancers, (liver, esophageal, breast, prostate, colon/rectal, leukemia, ovarian, uterine, pancreatic, kidney, gallbladder/biliary tract, and thyroid cancers) in SADC countries.

**Methods:**

Data from the 2019 Global Burden of Diseases Study was used. Deaths extracted from vital registration, verbal autopsies and ICD codes. Cancer‐type, mortality and prevalence per 100,000 population and 95% uncertainty intervals (UIs) were calculated using the Cause of Death Ensemble model and Spatio‐Temporal Gaussian process with mixed effects regression models. Annual rates of change (AROCs) between 1990 and 2019 and the corresponding UIs were calculated.

**Results:**

The top age‐standardized mortality rates per 100,000 in 2019 for males were leukemia, 20.1(14.4–26.4), esophageal cancer, 15.1 (11.2–19.1), and colon and rectal cancer, 10.3 (8.6–12.6). For females, breast cancer, 20.6 (16.6–25.0), leukemia, 17.1 (11.4–23.7), and esophageal cancer, 8.3 (5.5–10.7), had the leading mortality rates. For males, AROC substantial (*p* < 0.05) increase for kidney cancer for 11 of the countries (AROC from 0.41% to 1.24%), colon cancer for eight of the countries (from 0.39% to 0.92%), and pancreatic cancer for seven countries (from 0.26% to 1.01%). In females, AROC showed substantial increase for pancreatic cancer for 13 of the countries from (0.34%–1.67%), nine countries for kidney cancer (from 0.27% to 1.02%), seven countries each for breast cancer (0.35%–1.13%), and ovarian cancer (from 0.33% to 1.21%).

**Conclusions:**

There is need for location‐specific and culturally appropriate strategies for better nutrition and weight control, and improved screening for all cancers. Health promotion messaging should target kidney, colon, pancreatic, and breast cancers and encourage clinically tested methods of reducing BMI such as increasing personal physical activity and adoption of effective dietary regimes.

## Graphical abstract

Obesity‐related cancers in the 16 SADC countries is quite prominent but the time trends in mortality are unknown. We analyzed data obtained from the 2019 Global Burden of Diseases Study. For males in 2019, mortality rates for leukaemia, oesophageal, colon/rectum cancer were the most prominent, whereas for females, breast cancer, leukaemia, oesophageal cancers had the top mortality rates. Future studies should tease out the intersection of obesity, different types of cancers (especially for breast cancer in females, and leukaemia, oesophageal, colon/rectum cancer in both sexes), and risk factors in the environment such as industrial pollution, air and water pollution as well as climate change.

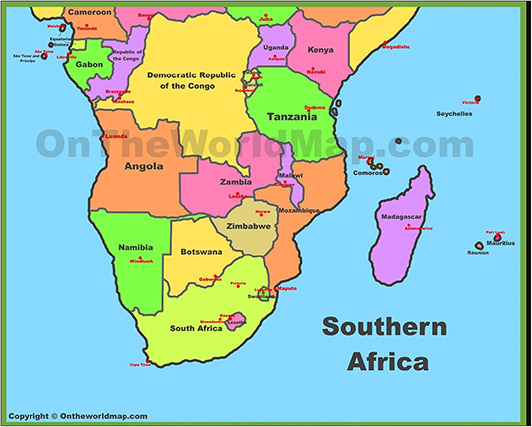

## 1 BACKGROUND

It has been projected that by 2030, 70% of cancer deaths may occur in developing countries, and that these countries are expected to bear the bulk of the projected 24.1 million new cases annually.^1^, ^2^ High body mass index (BMI exceeding 25 kg/m^2^) has been described as the largest health threat facing mankind today.^1^ Epidemiological studies have identified obesity as a risk factor for morbidity and mortality for many cancers such as esophagus, breast (in postmenopausal women), colon/rectal, uterus, gallbladder, kidney, liver, ovary, pancreas, and thyroid.^3^, ^4^, ^5^, ^6^, ^7^ A meta‐analysis of 57 prospective studies with 900,000 adults with over 6.5 million person‐years found that mortality was lowest for individuals with BMI within the normal range (22.5–25.0 kg/m^2^), and particularly for women.^8^ Each increase in BMI of 5 kg/m^2^ conferred a 29% increase in the hazard for all‐cause mortality, hazard ratio [HR] 1.29 (95% confidence interval [CI]: 1.27–1.32), and a 10% increase in the hazard for cancer mortality, 1.10, (1.06–1.15).^8^

A causal relationship has been established between overweight (BMI ≥30 kgs/m^2^) and obesity (BMI: 25‐<30 kg/m^2^) and cancer,^9^ but the role of increasing overweight and/or obesity on the trends of high‐BMI‐related cancers, particularly in developing countries, remains uncertain. Adults with obesity were reported to have a higher risk of cancer than those with a healthy weight in developed countries like the US.^6^, ^7^ Globally, an annual 0.6% increase was observed for the age‐standardized mortality rate and the burden of cancer attributable to obesity was heavier in regions with higher Socio‐Demographic Index (SDI) levels.^10^ The rate of incident cancers not associated with overweight and obesity decreased by 13%^6^, ^7^ while age‐adjusted rates of incident cancers associated with obesity [oesophagus, breast (in postmenopausal women), colon/rectal cancer; uterus, gallbladder, kidney, liver, ovary, pancreas, and thyroid cancers] in the US increased by 7% for the period 2005 to 2014. Currently, there is no such comparable data for many developing countries, especially those of the SADC. Goal 3.4 of the United Nations General Assembly 2030 agenda for Sustainable Development Goals (SDGs) aims to reduce by one third premature mortality from non‐communicable diseases (NCDs) with indicator 3.4.1 specifically seeking to reduce by one third the mortality rate attributed to conditions that are known to be causally associated with high BMI. Furthermore, epidemiologic analysis found “convincing or probable” evidence for a causal relationship between obesity^11^, ^12^ and 20 health outcomes including cancers of the oesophagus, colon/rectal, liver, gallbladder and biliary tract, pancreatic, breast, uterine, ovarian, thyroid and leukaemia.^13^, ^14^, ^15^, ^16^, ^17^, ^18^, ^19^, ^20^, ^21^, ^22^, ^23^, ^24^, ^25^, ^26^, ^27^, ^28^, ^29^, ^30^

Meta‐analyses relative risks (RR) of the association ranged from 1.2 to 1.5 for overweight, and from 1.5 to 1.8 for obesity for cancers of the colon/rectal,^13^, ^31^ gastric cardia,^32^ liver,^24^ gallbladder,^33^ pancreas^34^ and kidney cancers.^21^ The RR for oesophageal adenocarcinoma was as high as 4.8 for individuals with a BMI of 40 kg/m^2^ or more.^35^ However, 18 studies of the Asia Cohort Consortium found that the hazard ratios for oesophageal cancer and BMI formed a wide J‐shaped association indicating mortality risk increase for underweight (BMI < 18.5 kg/m^2^: HR = 2.20, 95% CI = 1.80–2.70) and extreme obesity (BMI ≥ 35 kg/m^2^: 4.38, 2.25–8.52) relative to the referent BMI category of (23–25 kg/m^2^).^36^ Other studies show substantial evidence of the positive association between increased BMI near the time of cancer diagnosis and reduced survival in patients with breast cancer^37^, ^38^, ^39^; however, recipients of bariatric surgery for intentional weight loss reported reductions in cancer incidence and mortality indicating a negative association between cancers and low BMI.^40^

**FIGURE 1:**
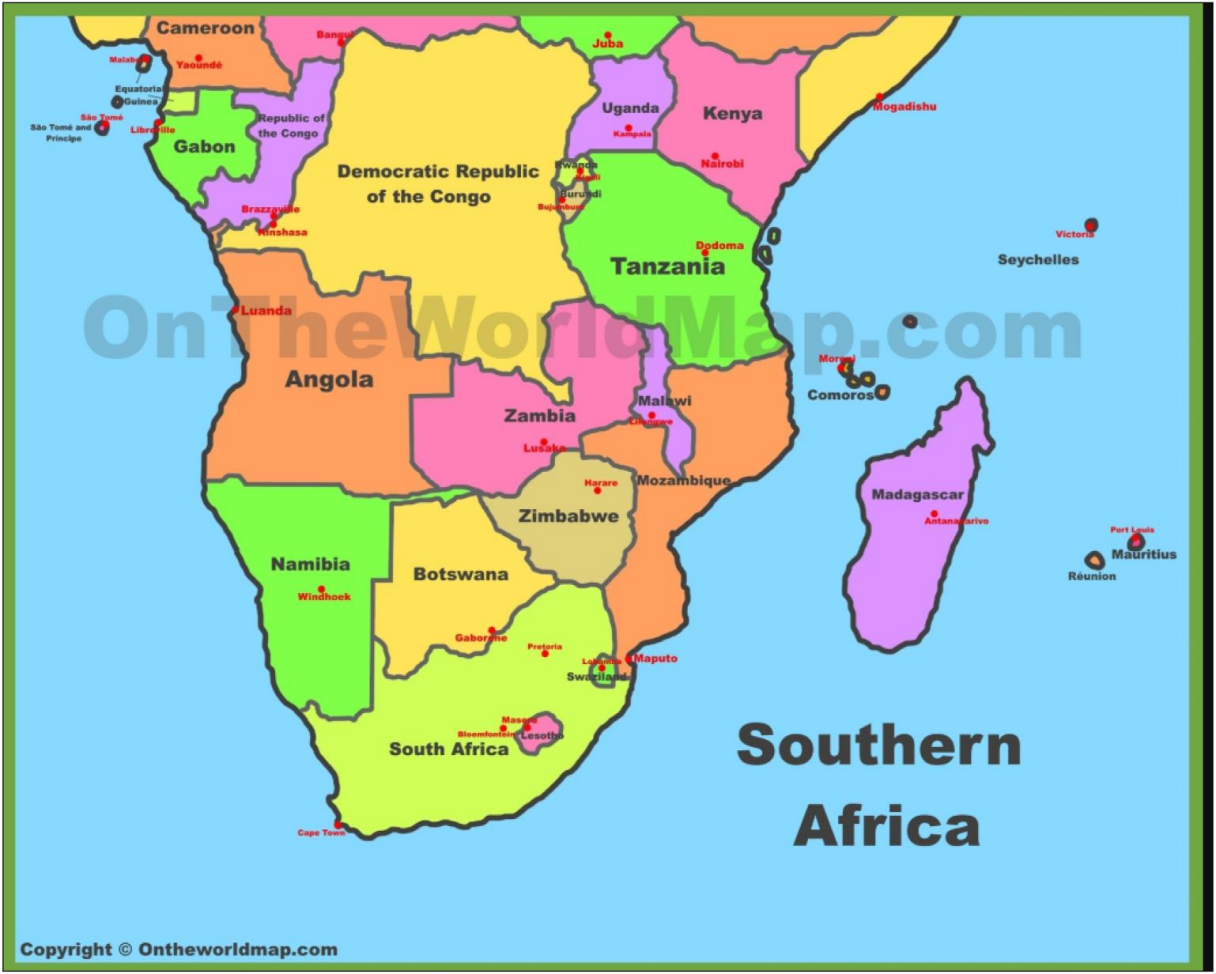
Map of Southern African development community countries. *Source*: http://ontheworldmap.com/africa/map-of-southern-africa.jpg.

Studies on cancer and BMI are extremely few in most developing countries.^41^, ^42^, ^43^ The 16 countries that form the Southern Africa Development Community (SADC) shown in Figure 1 (Angola, Botswana, Comoros, Democratic Republic of Congo [DRC], Lesotho, Madagascar, Malawi, Mauritius, Mozambique, Namibia, Seychelles, South Africa, Eswatini [formerly Swaziland], Tanzania, Zambia, and Zimbabwe) are no exception and they generally lack important information regarding the morbidity and mortality trends due to high‐BMI‐related cancers. As yet, there has not been any systematic understanding in SADC countries of the distribution of BMI related cancers and time trend analysis of the same. On the global scale, the International Agency for Research on Cancer's Global Cancer Observatory (GLOBOCAN) series estimated that there were 14.1 million new cancer cases and 8.2 million deaths worldwide in 2012 of which breast (1.67 million), and colorectal (1.36 million) were among the top three most commonly diagnosed, while liver (745,000 deaths), and stomach cancers (723,000 deaths) were among the top three most common causes of cancer death.^44^ Projection estimates from GLOBOCAN 2020 Study indicated that of the 34 cancer types studied, 1.1 million new cases (95% UIs: 1.0 – 1.3 million) and 711,429 (611,604 – 827,547) deaths due to neoplasms occurred in Africa in 2020, and that by 2040, the burden of all neoplasms combined is expected to increase to 2.1 million new cases and 1.4 million deaths in Africa alone.^45^ In 2019, for all countries of Africa, the Institute for Health Metrics and Evaluation (IHME) estimated a total of 71,708 breast cancer deaths, 48,639.97 deaths for colon/rectal cancer, 37,208 for liver cancer, 35,584.86 for oesophageal cancer 24,507 for pancreatic cancer, 17,624 for bladder cancer, 13,041 for ovarian cancer, 7366 for gallbladder and biliary tract cancer, 7201 for kidney cancer, 5901 for uterine cancer corresponding to age‐standardized mortality rates (95% uncertainty interval (UI)) ranging from 10.89 (9.53–12.34) per 100,000 population for breast cancer to 1.02 (0.80–1.22) for uterine cancer.^46^

Our previous report revealed that^47^ age‐standardized prevalence of overweight in adult females in SADC countries increased by 8.3% over a 30‐year period from 31.4% (30.5–32.3) in 1990 to 39.7% (38.7–40.7) in 2019; and increased in males by 8.5% from 20.2% (19.5–20.8) to 28.7% (27.9–29.5).^48^ Obesity in adult females increased by >1.5‐fold and nearly doubled in adult males. Despite these increasing trends in obesity and overweight, compounded by the well‐established causal relationship between obesity and several cancers, there is no known in‐depth analysis of trends in morbidity and mortality associated with high‐BMI‐related cancers for SADC countries. As a result, a descriptive epidemiological analysis of mortality and prevalence associated with high‐BMI‐related cancers for SADC countries was conducted using data from the Global Burden of Diseases (GBD), Injuries and Risk Factor Study.^47^ This information will aid key stakeholders to track progress and identify priorities for resource investment and/or develop corrective interventions to mitigate the interplay between obesity and related cancers.

## 2 METHODS

### 2.1 Data sources for cancer mortality

A description of GBD data sources and processing steps for the cause of death database, case definitions, input data for morbidity and mortality and modeling strategies for each cancer type are provided on pages 805 to 819 of Supplementary appendix 1 for GBD 2020 article.^47^ All data from GBD are anonymized and can be accessed on the website of the Institute for Health Metrics and Evaluation^47^ at the University of Washington Seattle. The GBD study uses deidentified, aggregated data and a waiver of informed consent approved by the University of Washington Institutional Review Board.

### 2.2 Input data

GBD obtained data from vital registration, verbal autopsies, and International Classification of Diseases (ICD) codes for the years 1990 and 2019.^49^ Cause of Death Ensemble model (CODEm) and Spatio‐Temporal Gaussian regression was used to estimate mortality due to individual cancer types. All ICD‐9 codes for cancer (140–209), and all ICD‐10 codes (C00‐C96, except for Kaposi's sarcoma (ICD‐10: C46) were included in the estimates for “malignant neoplasms”, specific details are described elsewhere.^50^

### 2.3 Estimation of cancer mortality

Studies providing national or sub‐national representative estimates for each type of cancer systematically searched on Medline are shown on Page 11 of the appendix, 2019. Search terms, selection criteria, and flow diagrams of screening and other details are provided elsewhere.^50^, ^51^ However, for illustration purposes, here is an example of the search description: to find the proportion of liver cancer cases (example) the following search string was used: “(“liver neoplasms” [All Fields] OR “HCC” [All Fields] OR “liver cancer” [All Fields] OR “Carcinoma, Hepatocellular” [Mesh]) AND ((“hepatitis B” [All Fields] OR “Hepatitis B” [Mesh] OR “Hepatitis B virus” [Mesh] OR “Hepatitis B Antibodies” [Mesh] OR “Hepatitis B Antigens” [Mesh]) OR (“hepatitis C”[All Fields] OR “Hepatitis C”[Mesh] OR “hepatitis C antibodies”[MESH] OR “Hepatitis C Antigens”[Mesh] OR “Hepacivirus”[Mesh]) OR (“alcohol”[All Fields] OR “Alcohol Drinking”[Mesh] OR “Alcohol‐Related Disorders” [Mesh] OR “Alcoholism”[Mesh] OR “Alcohol‐Induced Disorders”[Mesh])) NOT (animals [MeSH] NOT humans[MeSH])”.

### 2.4 Adjustment for country‐specific covariates

The CODEm modeling strategy for each cancer type was weighted for country‐specific covariates such as healthcare access and quality index (HAQI), education years per capita, SDI,^10^ age‐ and sex‐specific summary exposure variable for alcohol use, etc. A full description of coding of covariates and their influences can be found on pages 195 to 213 of Supplementary appendix 1 for GBD 2020 article^47^

### 2.5 Change in mortality rates over time

For each cancer type the slopes of the mortality rate from 1990 to 2019 were assessed using an annualized age‐standardized rate of change (AROC) as the percent difference in the natural logarithm of the rate in 1990 and 2019 divided by 30 (i.e., 100*[ln (2019 Rate/1990 Rate)/30]). AROC (%) represent a measure of a trend (increasing, decreasing or flat) over 30 years. A positive AROC indicates an increasing trend/slope or acceleration of mortality over the 30 years, and a negative AROC indicates decreasing mortality rate. To put negative AROC into perspective, two locations with different negative AROCs (e.g., −1.0% and −2.0%) indicates that the decline in mortality rate is less robust in the location with the lesser absolute AROC (−1.0%) than the decline in the location with the larger absolute AROC (−2.0%).

### 2.6 Uncertainty analysis

Uncertainty for each outcome was quantified using 95% uncertainty intervals (UIs) based on 1000 bootstrap draws from the posterior distribution.^52^, ^53^ UIs were determined by the 25th and 975th ordered values of the posterior distribution of the 1000 draws, and point estimates were computed from the mean. Changes over time were considered statistically significant when the 95% UI of the percentage change did not include zero.

#### 2.6.1 Reporting guidelines

The study did not require Institutional Review Board ethical review or informed consent as it used public GBD results and data. The GBD study followed the Strengthening the Reporting of Observational Studies in Epidemiology (STROBE) reporting guidelines (Elm E v 2007). In addition, all GBD estimates adhere to the 14 Guidelines on Accurate and Transparent Health Estimate Reporting (GATHER). GATHER recommends making available statistical code, why some sources are used and others are not, and how primary data are adjusted.^54^

#### 2.6.2 Role of the **funder**

The funders of this study had no role in the study design, data collection, data analysis, data interpretation, or the writing of the report. The corresponding author (PNG) had full access to the data in the study and final responsibility for the decision to submit for publication.

## 3 RESULTS

Our recent report found that obesity in adult females increased >1.5‐fold from 12.0% (95% UI: 11.5–12.4) in 1990 to 18.5% (17.9–19.0) in 2017; in adult males, obesity nearly doubled from 4.5% (4.3–4.8) to 8.8% (8.5–9.2). Mean BMI increased from 22.4 kg/m^2^ (21.6–23.1) to 23.1 kg/m^2^ (22.3–24.0) in adult males, and from 23.8 kg/m^2^ (22.9–24.7) to 24.8 kg/m^2^ (23.8–25.8) in adult females.^45^ Results presented in this paper should be considered in the above context.

### 3.1 Mortality rates

Table 1 shows age‐standardized mortality rates per 100,000 population due to the six leading cancers studied here (breast, oesophageal, colon/rectal, liver, prostate, and leukaemia).

**TABLE 1:** Age‐standardized mortality rate per 100,000 population due to 5 leading obesity‐related cancers in Southern Africa development community countries, 1990 and 2019.

	Breast		Esophageal		Colon and rectal		Liver		Leukemia	
	1990	2019	1990	2019	1990	2019	1990	2019	1990	2019
	Males	Males	Males	Males	Males	Males	Males	Males	Males	Males
Angola	0.6 (0.4–0.9)	0.6 (0.4–0.9)	15.8 (6.8–21.8)	12.3 (6.7–16.9)	9.5 (6.3–13.9)	11.7 (8.8–14.8)	3.6 (2.6–4.8)	3.6 (2.9–4.5)	29.2(12.3–74.8)	20.4(13.8–27.7)
Botswana	1.0 (0.7–1.4)	1.2 (0.8–1.6)	21.7 (12.1–30.4)	21.1 (13.4–27.6)	12.3 (9.4–15.8)	18.8 (13.9–23.4)	1.1 (0.4–3.7)	2.2 (1.6–3.0)	20.3(13.9–28.5)	25.7 (17.4–34.8)
Comoros	2.4 (1.3–3.8)	2.1 (1.1–3.5)	17.1 (8.6–24.4)	13.1 (8.7–19.0)	8.6 (5.5–11.4)	9.1 (6.5–12.4)	3.7 (1.7–7.9)	3.3 (2.0–6.6)	19.7 (8.9–31.5)	18.1 (10.0–30.9)
DRC	0.7 (0.4–1.6)	0.6 (0.3–1.5)	14.2 (6.5–20.3)	11.2 (6.0–16.9)	7.9 (5.7–14.0)	7.2 (4.5–14.2)	3.2 (2.6–4.0)	2.7 (2.0–3.7)	26.2 (13.8–55.0)	17.5 (12.2–25.1)
ESwatini	1.1 (0.7–1.6)	1.3 (0.7–2.1)	27.3 (14.9–37.3)	29.6 (18.9–39.4)	12.5 (9.0–17.1)	18.0 (11.5–25.2)	7.6 (4.1–19.8)	42.3 (9.8–76.2)	21.5 (15.0–27.4)	24.9 (16.6–34.8)
Lesotho	1.0 (0.6–1.4)	1.2 (0.8–1.8)	19.9 (10.9–26.9)	25.2 (16.3–32.0)	7.9 (5.9–11.8)	14.2 (10.4–19.0)	7.0 (3.6–20.0)	29.9 (10.3–50.5)	19.1 (13.6–24.7)	25.6 (17.4–35.4)
Madagascar	2.2 (1.3–3.4)	1.8 (1.0–2.9)	13.1 (6.7–18.9)	10.2 (6.5–15.4)	7.3 (5.8–8.9)	7.5 (5.2–10.5)	3.2 (1.5–7.7)	2.7 (1.4–5.3)	21.6 (14.0–36.3)	14.6 (8.7–22.3)
Malawi	2.9 (1.8–4.1)	2.7 (1.7–4.0)	25.9 (20.5–34.0)	34.4 (25.7–45.7)	5.2 (4.3–6.2)	7.3 (5.4–9.2)	3.9 (2.6–6.7)	4.2 (3.2–5.3)	18.3 (11.7–35.6)	13.2 (8.1–21.2)
Mauritius	0.1 (0.1–0.1)	0.1 (0.1–0.2)	4.3 (3.9–4.7)	3.7 (3.0–4.7)	9.0 (8.3–9.8)	15.5 (12.7–19.2)	2.4 (2.2–2.7)	2.8 (2.0–3.8)	39.1 (33.1–45.5)	30.2 (23.3–38.7)
Mozambique	0.1 (0.1–0.1)	0.1 (0.1–0.2)	10.7 (7.8–13.4)	14.8 (10.9–19.9)	5.4 (4.5–6.4)	10.4 (7.5–13.5)	2.6 (1.5–5.7)	6.4 (4.0–8.5)	27.1 (16.5–47.8)	28.1 (16.0–48.2)
Namibia	1.6 (1.1–2.4)	2.1 (1.4–2.8)	3.9 (2.9–5.1)	4.8 (3.6–6.2)	6.2 (4.7–7.8)	10.1 (8.2–12.3)	2.2 (1.1–5.3)	5.1 (3.9–6.5)	12.0 (8.7–15.5)	14.7 (10.1–19.8)
Seychelles	0.1 (0.1–0.2)	0.2 (0.2–0.3)	10.5 (8.5–12.4)	9.8 (8.1–11.9)	18.8 (15.9–21.5)	29.1 (24.7–34.4)	11.8 (9.9–14.3)	8.5 (6.8–10.4)	30.2 (25.2–37.6)	34.0 (26.9–42.7)
South Africa	0.6 (0.5–0.8)	0.6 (0.5–0.7)	20.7 (14.3–27.8)	16.5 (14.5–20.4)	11.6 (9.7–15.4)	14.0 (12.6–16.0)	7.5 (4.2–16.3)	9.2 (7.7–10.7)	24.6 (19.8–31.0)	21.9 (16.1–27.1)
Tanzania	3.1 (2.0–4.6)	2.6 (1.6–4.0)	18.2 (10.5–26.5)	14.4 (9.1–22.3)	8.8 (7.1–11.4)	10.4 (8.0–14.1)	2.5 (1.9–3.3)	2.9 (2.2–4.0)	26.1 (17.0–43.3)	24.4 (14.0–41.2)
Zambia	3.3 (2.1–4.8)	2.9 (1.8–4.7)	20.8 (11.8–26.2)	18.3 (12.4–25.6)	11.1 (8.7–13.8)	14.8 (10.9–18.8)	2.9 (1.8–5.0)	4.1 (3.2–5.1)	25.9 (15.3–48.6)	20.8 (13.0–32.7)
Zimbabwe	0.3 (0.2–0.4)	0.4 (0.3–0.5)	23.3 (19.4–27.7)	23.5 (18.3–30.8)	10.2 (8.8–11.7)	12.8 (9.8–15.7)	15.1 (10.3–27.7)	15.8 (10.9–25.3)	16.0 (11.9–19.7)	11.9 (8.6–17.9)
SADC	1.3 (1.0–1.7)	1.2 (0.9–1.6)	17.5 (11.4–22.1)	15.1 (11.2–19.1)	8.9 (7.6–10.8)	10.3 (8.6–12.6)	4.8 (3.4–8.4)	5.2 (4.6–5.8)	24.5 (16.8–41.2)	20.1 (14.4–26.4)

	1990	2019	1990	2019	1990	2019	1990	2019	1990	2019
	Females	Females	Females	Females	Females	Females	Females	Females	Females	Females
Angola	14.0 (9.9–18.6)	20.0 (15.0–26.3)	8.0 (3.9–11.6)	6.0 (2.9–8.7)	6.6 (4.1–10.2)	8.1 (6.3–10.4)	2.0 (1.4–2.9)	1.8 (1.4–2.4)	29.5 (10.0–70.4)	15.0 (9.3–22.2)
Botswana	19.3 (14.1–26.2)	28.6 (19.0–41.0)	9.0 (4.5–12.9)	7.6 (3.9–10.9)	9.3 (6.8–12.6)	13.5 (9.7–19.0)	0.9 (0.5–1.4)	1.0 (0.7–1.4)	13.0 (9.1–17.3)	25.3 (15.9–37.1)
Comoros	14.4 (7.8–20.4)	19.7 (14.8–25.8)	13.3 (7.2–18.4)	11.0 (6.9–15.0)	7.2 (4.5–10.3)	8.2 (5.9–10.7)	3.0 (1.9–4.2)	2.8 (2.0–3.6)	20.0 (9.0–32.8)	17.1 (9.3–30.4)
DRC	18.0 (14.1–22.3)	22.5 (15.8–30.6)	8.1 (3.5–11.5)	6.8 (3.2–10.3)	5.9 (4.5–8.0)	5.5 (3.5–9.0)	2.2 (1.7–2.7)	2.0 (1.5–2.7)	22.8 (9.8–47.3)	11.7 (6.3–19.3)
ESwatini	16.6 (11.8–21.1)	23.0 (14.6–33.5)	8.7 (4.4–12.6)	7.1 (3.8–10.4)	8.5 (6.5–10.9)	10.6 (7.0–14.7)	4.6 (3.2–6.6)	4.7 (2.7–7.8)	13.9 (9.4–18.8)	14.9 (9.6–21.5)
Lesotho	13.3 (10.2–17.6)	28.4 (18.1–41.8)	7.1 (3.8–9.3)	8.9 (4.7–12.7)	5.5 (4.0–8.0)	9.9 (6.6–13.6)	4.2 (2.9–6.8)	5.7 (3.4–9.2)	9.9 (6.3–13.9)	15.1 (9.5–22.8)
Madagascar	14.2 (11.6–16.9)	16.8 (12.3–22.4)	11.9 (6.5–15.1)	10.4 (6.2–14.8)	6.3 (4.7–7.9)	6.8 (5.0–9.4)	2.9 (2.3–3.7)	2.5 (1.8–3.4)	23.9 (12.6–38.1)	13.2 (8.1–20.7)
Malawi	13.3 (10.3–16.2)	17.2 (13.2–21.7)	20.3 (16.2–25.5)	18.4 (13.6–24.9)	4.4 (3.5–5.2)	5.1 (3.9–6.6)	3.0 (2.4–3.8)	2.2 (1.7–2.8)	20.9 (10.2–35.7)	11.9 (7.0–20.8)
Mauritius	12.1 (11.1–13.1)	22.0 (17.7–27.1)	2.6 (2.4–2.8)	1.5 (1.3–1.9)	7.5 (6.9–8.2)	10.6 (8.7–12.8)	1.5 (1.3–1.6)	1.4 (1.1–1.7)	38.4 (32.8–44.9)	29.4 (23.0–37.2)
Mozambique	14.9 (11.9–18.3)	21.5 (15.5–29.4)	3.3 (2.1–4.2)	3.4 (2.1–4.8)	4.9 (3.8–6.1)	7.4 (5.3–10.1)	2.0 (1.5–2.7)	2.3 (1.6–3.2)	34.4 (14.3–64.8)	26.8 (14.3–53.4)
Namibia	16.7 (12.7–20.9)	29.7 (20.9–41.7)	1.5 (1.1–1.8)	1.2 (0.9–1.7)	5.3 (4.1–6.8)	7.6 (5.6–10.0)	1.7 (1.3–2.3)	2.2 (1.6–3.0)	9.8 (5.9–13.9)	12.4 (8.2–17.4)
Seychelles	15.4 (13.7–17.4)	25.0 (20.7–29.7)	2.1 (1.7–2.4)	1.9 (1.5–2.3)	14.7 (12.9–16.7)	21.7 (18.2–25.6)	4.8 (4.0–5.7)	3.1 (2.4–3.7)	32.2 (26.3–40.8)	42.7 (33.6–55.1)
South Africa	18.8 (16.3–22.1)	20.5 (18.1–23.2)	8.8 (6.2–11.1)	6.5 (5.5–7.9)	9.4 (7.6–11.7)	9.3 (8.1–10.6)	4.9 (3.5–6.4)	3.5 (2.9–4.5)	19.6 (15.4–23.6)	16.0 (13.4–18.6)
Tanzania	13.2 (10.6–15.7)	16.8 (12.3–22.4)	13.2 (7.1–17.1)	10.7 (6.6–15.0)	7.1 (5.7–8.6)	8.8 (7.2–10.6)	2.0 (1.6–2.4)	2.0 (1.6–2.5)	28.5 (13.8–49.9)	26.4 (13.7–44.6)
Zambia	17.9 (14.4–22.1)	19.5 (14.8–25.7)	16.1 (9.1–21.3)	11.6 (7.2–16.2)	9.6 (6.3–13.3)	10.7 (7.7–14.4)	2.2 (1.7–3.0)	2.1 (1.6–2.7)	31.2 (14.3–55.3)	19.2 (11.4–31.8)
Zimbabwe	17.7 (14.7–21.1)	26.9 (18.7–37.2)	8.3 (6.6–10.1)	10.7 (7.2–14.7)	9.5 (8.1–11.4)	12.9 (9.5–17.1)	9.2 (7.4–11.3)	12.6 (8.7–18.0)	12.8 (9.4–16.6)	11.3 (8.1–15.6)
SADC	16.3 (14.4–18.1)	20.6 (16.6–25.0)	9.9 (6.2–11.8)	8.3 (5.5–10.7)	7.2 (6.1–8.2)	7.9 (6.6–9.4)	3.2 (2.8–3.8)	2.8 (2.3–3.3)	23.7 (13.7–36.7)	17.1 (11.4–23.7)

*Note*: Cancer‐type, age‐standardized mortality rates and 95% uncertainty intervals (UIs) were calculated for each country using the Cause of Death Ensemble model 22 (Foremans, 2012), Vander Hoorn S, 2004).

Abbreviations: BMI, body mass index (kg/m^2^); DRC, Democratic Republic of the Congo.

#### 3.1.1 Breast cancer

In all 16 countries combined, female breast cancer had the highest mortality rate of 16.3 (95% UI: 14.4–18.1) in 1990, increasing to 20.6 (16.6–25.0) in 2019. The mortality rate for female breast cancer in 2019 was 1.26‐fold higher than of the rate in 1990. In 2019, female breast cancer mortality rate (95% UI) of 29.7 (20.9–41.7) was the highest in Namibia followed by Botswana 28.6 (19.0–41.0) and Lesotho 28.4 (18.1–41.8). Relative to the SADC, the 2019 female breast cancer mortality rate was 1.44‐fold higher than 1990 for Namibia, 1.39‐fold higher for Botswana, and 1.38‐fold higher for Lesotho.

#### 3.1.2 Oesophageal cancer

The second most common type of cancer among males was oesophageal cancer with mortality rate (95% UI) at 17.5 (11.4–22.1) in 1990, decreasing to 15.1 (11.2–19.1) in 2019; in females, oesophageal mortality rate was 9.9 (6.2–11.8) in 1990, decreasing to 8.3 (5.5–10.7) in 2019. For individual SADC countries, oesophageal cancer mortality in 2019 was highest in Malawi, with a rate of 34.4 (25.7–45.7) for males but only one half the corresponding mortality rate for females 18.4 (13.6–24.9).

#### 3.1.3 Colon and rectal cancer

The highest mortality rate (95% UI) due to cancer of the colon/rectal among males was observed in Seychelles, 29.1 (24.7–34.4), followed by Botswana, 18.8 (13.9–23.4), and Eswatini 18.0 (11.5–25.2). In females, Seychelles, 21.7 (18.2–25.6), Botswana, 13.5 (9.7–19.0), and Zimbabwe 12.9 (9.5–17.1) had the highest mortality rates for colon/rectal cancer.

#### 3.1.4 Liver cancer

The combined SADC mortality rate (95% UI) for liver cancer among males increased from 4.8 (3.4–8.4) in 1990 to 5.2 (4.6–5.8) in 2019; however, liver cancer mortality rate for females decreased from 3.2 (2.8–3.8) to 2.8 (2.3–3.3) during the same period. In 2019, liver cancer mortality rates were highest (double digit) in Eswatini 42.3 (9.8–76.2), Lesotho 29.9 (10.3–50.5), and Zimbabwe 15.8 (10.9–25.3) among males; among females however, only Zimbabwe, 12.6 (8.7–18.0) had a double‐digit liver cancer mortality in 2019. It is important to note that the 2019 liver cancer mortality rate for males in Eswatini and Lesotho were 8.1 and 5.8 times, respectively relative to the combined SADC rate of 5.2 (4.6–5.8). In females, the 2019 liver cancer mortality rate for Zimbabwe was 4.5 times the combined SADC rate of 12.6 (8.7–18.0).

#### 3.1.5 Leukaemia

In 2019, leukaemia mortality rate (95% UI) for males was 20.1 (14.4–26.4) while that of females was 17.1 (11.4–23.7). Seychelles had the highest death rate of 34.0 (26.9–42.7) for males and 42.7 (33.6–55.1) for females respectively; Mauritius followed with death rates of 30.2 (23.3–38.7) for males and 29.4 (23.0–37.2) for females respectively.

### 3.2 Age‐standardized annual rate of change

Table 2 shows age‐standardized AROC% between 1990 and 2019 for obesity related cancers. For males, age‐standardized AROC showed statistically significant (*p* < 0.05) linear increase for kidney cancer for 11 of the countries (AROC from 0.41% to 1.24%). The two countries with the steepest AROC increase for kidney cancer were Lesotho 1.24(0.56; 2.38), and Mozambique 0.98(0.23; 2.26). AROC for colon/rectal cancer for males was statistically significant for eight of the countries (from 0.39% to 0.92%) with the Mozambique 0.92(0.29; 1.68) and Lesoth0 0.80(0.18; 1.50) leading. AROC for pancreatic cancer for seven countries (from 0.26% to 1.01%), with Namibia 1.01(0.46; 1.90) and Mozambique 0.95(0.24; 2.15) ranked first and second, respectively. For females, age‐standardized AROC showed statistically significant linear increase for pancreatic cancer for 13 of the countries from (0.34%–1.67%), nine countries for kidney cancer (from 0.27% to 1.02%), seven countries each for breast cancer (0.35%–1.13%), and ovarian cancer (from 0.33% to 1.21%). The three leading countries with the top AROC increase for pancreatic cancer were Lesotho, 1.67(0.50; 3.42), Botswana 1.37(0.55; 2.59), and Namibia 1.37(0.59; 2.43). The two countries with the steepest AROC for kidney cancer for females were Lesotho 1.02(0.27; 2.27), and Tanzania 0.74 (0.28; 1.31). AROC for breast cancer was steepest in Lesotho 1.13(0.24; 2.44) followed by Mauritius, 0.82(0.44; 1.29), whereas ovarian cancer had the steepest slope in Lesotho, 1.21(0.19; 3.26) followed by Botswana, 0.77(0.05; 1.80).

**TABLE 2:** Age‐standardized annual rates of change (AROC) % for obesity‐related cancers in Southern Africa development community countries, 1990 and 2019.

	Males										
	Breast	Esophageal	Colon & rectal	Liver	Leukemia	Uterine cancer	Ovarian	Pancreatic	Kidney	Gallbladder/biliary tract	Thyroid
Angola	0.03 (−0.39; 0.74)	−0.22 (−0.45; 0.19)	0.23 (−0.18; 0.85)	0.02 (−0.33; 0.52)	−0.15 (−0.46; 0.40)	‐	‐	0.45 (−0.15; 1.45)	0.47 (−0.10; 1.50)	−0.06 (−0.38; 0.48)	0.05 (−0.30; 0.63)
Botswana	0.14 (–0.30; 0.83)	−0.03 (−0.33; 0.37)	0.52 (0.10; 1.03)	0.94 (−0.36; 4.52)	0.08 (−0.23; 0.46)	‐	‐	0.83 (0.32; 1.49)	0.88 (0.28; 1.96)	0.06 (‐0.21; 0.41)	0.42 (−0.02; 1.01)
Comoros	−0.12 (−0.48; 0.54)	−0.24 (−0.49; 0.40)	0.05 (−0.33; 0.77)	−0.09 (−0.38; 0.60)	0.01 (−0.36; 0.92)	‐	‐	0.20 (−0.23; 1.04)	0.31 (−0.23; 1.33)	−0.04 (−0.32; 0.56)	0.26 (−0.14; 1.33)
DRC	−0.11 (−0.48; 0.41)	−0.21 (−0.42; 0.09)	−0.08 (−0.44; 0.36)	−0.15 (−0.41; 0.17)	−0.16 (−0.42; 0.22)	‐	‐	0.06 (−0.32; 0.49)	0.03 (−0.45; 0.56)	−0.16 (−0.43; 0.13)	−0.08 (−0.36; 0.22)
ESwatini	0.18 (−0.34; 0.98)	0.09 (−0.22; 0.49)	0.44 (0.04; 0.96)	4.54 (−0.40; 13.79)	0.16 (−0.13; 0.53)	‐	‐	0.77 (0.26; 1.53)	0.92 (0.34; 1.81)	0.13 (−0.14; 0.50)	0.61 (0.16; 1.24)
Lesotho	0.30 (−0.23; 1.13)	0.26 (−0.08; 0.74)	0.80 (0.18; 1.50)	3.27 (−0.34; 9.08)	0.42 (0.04; 0.89)			0.92 (0.20; 1.93)	1.24 (0.56; 2.38)	0.33 (0.03; 0.77)	0.77 (0.28; 1.46)
Madagascar	−0.19 (−0.52; 0.30)	−0.22 (−0.46; 0.13)	0.02 (−0.30; 0.41)	−0.15 (−0.43; 0.30)	−0.14 (−0.41; 0.22)	‐	‐	0.13 (−0.21; 0.55)	0.23 (−0.19; 0.76)	−0.05 (−0.34; 0.33)	0.23 (−0.13; 0.72)
Malawi	−0.06 (−0.43; 0.50)	0.33 (0.01; 0.79)	0.39 (0.00; 0.93)	0.07 (−0.35; 0.61)	−0.10 (−0.40; 0.33)	‐	‐	0.46 (−0.02; 1.21)	0.38 (−0.10; 1.15)	0.07 (−0.17; 0.39)	0.22 (−0.12; 0.69)
Mauritius	1.09 (0.62; 1.66)	−0.13 (−0.32; 0.10)	0.72 (0.37; 1.11)	0.17 (−0.19; 0.68)	−0.10 (−0.28; 0.10)	‐	‐	0.27 (0.00; 0.57)	0.77 (0.41; 1.19)	−0.43 (−0.55; −0.22)	−0.08 (−0.28; 0.18)
Mozambique	0.42 (−0.11; 1.30)	0.39 (0.01; 1.03)	0.92 (0.29; 1.68)	1.46 (−0.31; 3.77)	0.15 (−0.17; 0.68)	‐	‐	0.95 (0.24; 2.15)	0.98 (0.23; 2.26)	0.40 (0.07; 0.88)	0.93 (0.34; 1.78)
Namibia	0.26 (−0.22; 1.05)	0.24 (‐0.08; 0.72)	0.61 (0.20; 1.23)	1.27 (−0.07; 4.03)	0.11 (−0.17; 0.55)	‐	‐	1.01 (0.46; 1.90)	0.84 (0.31; 1.85)	0.09 (−0.19; 0.51)	0.63 (0.12; 1.28)
Seychelles	0.88 (0.26; 1.81)	−0.06 (−0.25; 0.19)	0.55 (0.27; 0.90)	−0.28 (−0.45; −0.08)	−0.08 (−0.26; 0.13)	‐	‐	0.26 (0.00; 0.56)	0.75 (0.38; 1.24)	−0.23 (−0.41; 0.05)	−0.05 (−0.27; 0.22)
South Africa	0.03 (−0.16; 0.27)	−0.21 (−0.39; 0.16)	0.20 (−0.02; 0.46)	0.22 (−0.51; 1.43)	−0.03 (−0.20; 0.12)	‐	‐	0.31 (0.07; 0.58)	0.41 (0.10; 0.68)	0.04 (−0.16; 0.21)	0.20 (0.05; 0.37)
Tanzania	−0.17 (−0.48; 0.37)	−0.21 (−0.42; 0.08)	0.18 (−0.09; 0.49)	0.19 (−0.13; 0.61)	0.01 (−0.33; 0.48)	‐	‐	0.28 (−0.08; 0.74)	0.57 (0.05; 1.82)	0.00 (−0.24; 0.34)	0.35 (−0.05; 0.86)
Zambia	−0.09 (−0.45; 0.47)	−0.12 (−0.36; 0.26)	0.34 (−0.02; 0.82)	0.42 (−0.23; 1.28)	−0.05 (−0.33; 0.41)	‐	‐	0.40 (−0.06; 1.05)	0.56 (0.05; 1.37)	0.07 (−0.22; 0.42)	0.48 (0.06; 1.16)
Zimbabwe	0.22 (−0.20; 0.87)	0.00 (−0.23; 0.29)	0.25 (−0.07; 0.64)	0.05 (−0.28; 0.45)	−0.09 (−0.35; 0.29)	‐	‐	0.34 (0.00; 0.75)	0.47 (0.08; 1.02)	−0.19 (−0.43; 0.21)	0.28 (−0.03; 0.65)
Females											
Angola	0.42 (–0.03; 1.07)	−0.25 (−0.50; 0.14)	0.23 (−0.21; 1.00)	−0.10 (−0.42; 0.40)	−0.24 (−0.55; −0.46)	−0.03 (−0.34; 0.50)	0.64 (0.01; 1.63)	0.93 (0.26; 1.89)	0.51 (−0.03; 1.36)	−0.09 (−0.40; 0.54)	−0.03 (−0.34; 0.49)
Botswana	0.48 (−0.10; 1.30)	−0.16 (−0.46; 0.31)	0.45 (−0.02; 1.14)	0.10 (−0.39; 0.98)	0.27 (−0.23; −0.91	0.33 (−0.18; 1.01)	0.77 (0.05; 1.80)	1.37 (0.55; 2.59)	0.66 (0.02; 1.83)	0.03 (−0.34; 0.62)	0.05 (−0.39; 0.66)
Comoros	0.36 (−0.11; 1.49)	−0.18 (−0.43; 0.37)	0.14 (−0.30; 0.93)	−0.07 (−0.39; 0.55)	0.01 (−0.37; 1.07)	0.01 (−0.33; 0.66)	0.83 (−0.08; 2.65)	0.54 (0.11; 1.40)	0.59 (−0.09; 1.70)	0.04 (−0.31; 0.75)	0.26 (−0.19; 1.31)
DRC	0.25 (−0.13; 0.77)	−0.16 (−0.39; 0.17)	−0.07 (−0.42; 0.36)	−0.07 (−0.35; 0.34)	−0.24 (−0.54; 0.25)	−0.06 (−0.36; 0.34)	0.32 (−0.18; 1.21)	0.22 (−0.18; 0.71)	0.08 (−0.35; 0.52)	−0.12 (−0.44; 0.24)	−0.06 (−0.32; 0.26)
ESwatini	0.39 (−0.16; 1.32)	−0.19 (−0.51; 0.25)	0.25 (−0.19; 0.86)	0.02 (−0.47; 1.15)	0.07 (−0.32; 0.54)	0.14 (−0.31; 0.75)	0.43 (−0.23; 1.61)	0.67 (0.00; 1.64)	0.36 (−0.14; 1.13)	−0.02 (−0.38; 0.48)	−0.02 (−0.40; 0.47)
Lesotho	1.13 (0.24; 2.44)	0.26 (−0.22; 0.83)	0.81 (0.05; 1.85)	0.36 (−0.29; 1.84)	0.68 (0.02; 1.51)	0.89 (0.19; 1.78)	1.21 (0.19; 3.26)	1.67 (0.50; 3.42)	1.02 (0.27; 2.27)	0.46 (−0.05; 1.16)	0.49 (−0.11; 1.25)
Madagascar	0.18 (−0.16; 0.64)	−0.13 (−0.39; 0.20)	0.08 (−0.26; 0.54)	−0.13 (−0.37; 0.20)	−0.17 (−0.42; 0.28)	0.01 (−0.27; 0.43)	0.32 (−0.17; 1.06)	0.34 (−0.05; 0.82)	0.34 (−0.08; 0.91)	0.08 (−0.26; 0.52)	0.18 (−0.20; 0.66)
Malawi	0.30 (−0.07; 0.81)	−0.09 (−0.34; 0.27)	0.18 (−0.18; 0.60)	−0.27 (−0.47; −0.02)	−0.21 (−0.49; 0.32)	−0.12 (−0.38; 0.24)	0.50 (−0.25; 1.56)	0.58 (0.13; 1.12)	0.34 (−0.19; 0.93)	−0.17 (−0.41; 0.26)	−0.02 (−0.32; 0.38)
Mauritius	0.82 (0.44; 1.29)	0.40 (−0.52; −0.25)	0.41 (0.15; 0.75)	−0.09 (−0.27; 0.16)	−0.11 (−0.28; 0.10)	−0.29 (−0.44; −0.12)	0.65 (0.29; 1.11)	0.22 (−0.04; 0.52)	0.45 (0.15; 0.79)	−0.22 (−0.40; −0.01)	−0.21 (−0.37; −0.01)
Mozambique	0.45 (0.03; 1.00)	0.04 (−0.29; 0.48)	0.50 (0.06; 1.16)	0.12 (−0.26; 0.68)	−0.04 (−0.42; 0.52)	0.11 (−0.24; 0.62)	0.51 (−0.02; 1.27)	1.15 (0.40; 2.07)	0.65 (0.13; 1.37)	0.17 (−0.22; 0.68)	0.30 (−0.15; 0.89)
Namibia	0.77 (0.19; 1.65)	−0.15 (−0.42; 0.23)	0.43 (−0.01; 1.05)	0.29 (−0.18; 1.03)	0.06 (−0.30; 0.60)	0.06 (−0.28; 0.50)	0.42 (−0.12; 1.21)	1.37 (0.59; 2.43)	0.48 (0.00; 1.20)	−0.05 (−0.35; 0.42)	0.02 (−0.32; 0.51)
Seychelles	0.62 (0.31; 0.99)	−0.11 (−0.30; 0.13)	0.48 (0.20; 0.81)	−0.37 (−0.52; −0.18)	−0.17 (−0.34; 0.05)	0.03 (−0.18; 0.30)	0.60 (0.11; 1.10)	0.50 (0.21; 0.87)	0.27 (0.00; 0.61)	−0.17 (−0.35; 0.13)	−0.13 (−0.32; 0.11)
South Africa	0.09 (−0.05; 0.26)	−0.27 (−0.38; 0.01)	−0.01 (−0.15; 0.20)	−0.28 (−0.46; −0.05)	−0.17 (−0.27; −0.03)	0.29 (0.07; 0.49)	0.33 (0.06; 0.55)	0.34 (0.10; 0.58)	0.05 (−0.08; 0.20)	−0.04 (−0.15; 0.07)	−0.10 (−0.21; 0.04)
Tanzania	0.35 (0.03; 0.79)	−0.19 (−0.37; 0.06)	0.24 (−0.02; 0.58)	0.04 (−0.22; 0.36)	0.07 (−0.28; 0.63)	0.04 (−0.20; 0.36)	0.43 (−0.01; 1.11)	0.58 (0.29; 0.99)	0.74 (0.28; 1.31)	0.07 (−0.18; 0.44)	0.23 (−0.12; 0.66)
Zambia	0.09 (−0.23; 0.50)	−0.28 (−0.49; 0.06)	0.12 (−0.26; 0.61)	−0.07 (−0.39; 0.38)	−0.18 (−0.47; 0.38)	−0.11 (−0.36; 0.26)	0.39 (−0.18; 1.28)	0.51 (0.09; 1.03)	0.53 (0.05; 1.22)	−0.07 (−0.36; 0.38)	0.07 (−0.30; 0.64)
Zimbabwe	0.52 (0.05; 1.16)	0.29 (−0.12; 0.83)	0.35 (−0.07; 0.89)	0.36 (−0.08; 0.98)	0.01 (−0.28; 0.40)	0.49 (0.01; 1.07)	0.68 (0.05; 1.52)	0.76 (0.22; 1.43)	0.44 (0.01; 1.00)	0.02 (−0.28; 0.39)	0.22 (−0.14; 0.75)

*Note*: AROC % was calculated as the percent difference in the natural logarithm of the rate in 1990 and 2019 divided by 30 (i.e., 100*[ln(2019 Rate/1990 Rate)/30]).

Abbreviations: ‐, Not applicable for sex; AROC %, annualized rate of change (percent); BMI, body mass index (kg/m^2^); DRC, Democratic Republic of the Congo.

Table 3 shows age‐standardized mortality rates for other high‐BMI‐related cancers (ovarian, uterine, pancreatic, kidney, gallbladder and biliary tract, and thyroid) for 1990 and 2019. The combined SADC uterine cancer mortality rate increased from 3.0 (2.3–4.1) in 1990 to 4.2 (3.4–5.2) in 2019 corresponding to a 2019/1990 mortality rate ratio of 1.40 (1.26–1.47). Fourteen of the 16 SADC countries had uterine cancer mortality rates greater than 3.0 in 2019, four having greater than 6.0, with the top mortality rates recorded in Seychelles, 8.9 (7.0–10.8), and Zimbabwe, 7.5 (5.2–10.1). Fifteen countries had ovarian cancer mortality exceeding 2.0, six exceeding 3.0. Zimbabwe, 5.5 (3.3–7.5), Lesotho 3.9 (2.5–5.7), and Botswana 3.7 (2.4–5.3).

**TABLE 3:** Age‐standardized mortality rate per 100,000 population due to other obesity‐related cancers in Southern Africa development community countries, 1990 and 2019.

	Uterine cancer		Ovarian		Pancreatic		Kidney		Gallbladder/biliary tract		Thyroid	
	1990	2019	1990	2019	1990	2019	1990	2019	1990	2019	1990	2019
	Males	Males	Males	Males	Males	Males	Males	Males	Males	Males	Males	Males
Angola	‐	‐			2.1 (1.4–3.0)	3.1 (2.3–4.0)	1.1 (0.7–1.6)	1.6 (1.0–2.5)	1.2 (0.8–1.6)	1.1 (0.8–1.6)	0.3 (0.2–0.5)	0.3 (0.3–0.5)
Botswana	‐	‐	‐	‐	3.3 (2.5–4.2)	6.3 (4.4–8.4)	1.2 (0.8–1.6)	2.2 (1.7–2.8)	1.0 (0.8–1.4)	1.1 (0.8–1.6)	0.1 (0.1–0.1)	0.1 (0.1–0.2)
Comoros	‐	‐	‐	‐	2.4 (1.3–3.2)	2.9 (1.9–4.1)	1.3 (0.7–1.7)	1.6 (1.1–2.4)	0.9 (0.6–1.3)	0.9 (0.6–1.2)	0.4 (0.2–0.5)	0.5 (0.3–0.7)
DRC	‐	‐	‐	‐	2.0 (1.4–2.7)	2.1 (1.4–2.9)	1.0 (0.7–1.5)	1.0 (0.5–1.8)	1.1 (0.8–1.4)	0.9 (0.5–1.2)	0.3 (0.2–0.4)	0.3 (0.2–0.4)
ESwatini	‐	‐	‐	‐	4.6 (3.0–6.8)	8.2 (5.1–12.3)	1.7 (1.1–2.4)	3.2 (2.1–4.9)	1.1 (0.8–1.4)	1.2 (0.9–1.6)	0.4 (0.3–0.5)	0.7 (0.4–0.9)
Lesotho	‐	‐	‐	‐	2.7 (1.9–3.7)	5.1 (3.7–7.2)	0.9 (0.5–1.4)	2.0 (1.3–2.8)	0.9 (0.7–1.2)	1.2 (0.9–1.6)	0.3 (0.2–0.4)	0.5 (0.3–0.6)
Madagascar	‐	‐	‐	‐	1.6 (1.2–2.1)	1.8 (1.1–2.7)	0.9 (0.6–1.3)	1.1 (0.7–1.7)	0.8 (0.6–1.0)	0.7 (0.5–1.0)	0.3 (0.2–0.4)	0.4 (0.2–0.6)
Malawi	‐	‐	‐	‐	1.6 (1.2–2.0)	2.3 (1.6–3.2)	2.3 (1.8–2.9)	3.1 (2.1–4.4)	0.7 (0.5–0.9)	0.8 (0.6–1.0)	0.3 (0.2–0.3)	0.3 (0.2–0.4)
Mauritius	‐	‐	‐	‐	3.8 (3.5–4.1)	5.0 (3.9–6.1)	1.1 (1.0–1.1)	1.9 (1.5–2.3)	1.6 (1.2–1.8)	0.9 (0.7–1.2)	0.3 (0.3–0.3)	0.3 (0.2–0.4)
Mozambique	‐	‐	‐	‐	1.5 (1.1–1.8)	2.9 (2.0–4.2)	0.7 (0.5–0.9)	1.4 (0.9–2.0)	1.0 (0.7–1.4)	1.4 (1.0–1.9)	0.4 (0.3–0.5)	0.7 (0.5–1.0)
Namibia	‐	‐	‐	‐	1.5 (1.1–1.8)	3.0 (2.3–3.8)	1.3 (0.9–1.6)	2.3 (1.7–3.0)	0.6 (0.5–0.8)	0.7 (0.5–0.9)	0.2 (0.2–0.3)	0.4 (0.3–0.5)
Seychelles	‐	‐	‐	‐	5.6 (4.6–6.5)	7.2 (6.0–8.6)	1.7 (1.4–2.0)	2.9 (2.3–3.5)	1.7 (1.2–2.1)	1.3 (1.0–1.6)	0.4 (0.3–0.4)	0.3 (0.3–0.4)
South Africa	‐	‐	‐	‐	4.1 (3.4–5.4)	5.3 (4.6–6.1)	1.5 (1.3–1.8)	2.1 (1.8–2.3)	0.7 (0.6–1.0)	0.7 (0.6–0.9)	0.3 (0.2–0.3)	0.3 (0.3–0.4)
ESwatini	‐	‐	‐	‐	4.6 (3.0–6.8)	8.2 (5.1–12.3)	1.7 (1.1–2.4)	3.2 (2.1–4.9)	1.1 (0.8–1.4)	1.2 (0.9–1.6)	0.4 (0.3–0.5)	0.7 (0.4–0.9)
Tanzania	‐	‐	‐	‐	2.3 (1.8–3.0)	3.0 (2.2–4.4)	1.2 (0.9–1.7)	1.9 (1.3–3.3)	1.0 (0.8–1.4)	1.0 (0.7–1.5)	0.4 (0.3–0.5)	0.5 (0.4–0.8)
Zambia	‐	‐	‐	‐	2.8 (2.3–3.5)	4.1 (2.9–5.8)	1.5 (0.9–2.1)	2.4 (1.3–3.6)	1.1 (0.9–1.4)	1.2 (0.9–1.5)	0.5 (0.4–0.6)	0.7 (0.5–0.9)
Zimbabwe	‐	‐	‐	‐	3.4 (2.9–3.9)	4.6 (3.4–5.8)	0.8 (0.7–1.0)	1.2 (0.9–1.6)	1.5 (1.1–1.7)	1.2 (0.9–1.8)	0.4 (0.4–0.5)	0.6 (0.5–0.7)
SADC					2.6 (2.2–3.0)	3.2 (2.6–3.8)	1.2 (1.0–1.4)	1.6 (1.3–2.1)	1.0 (0.8–1.1)	1.0 (0.8–1.2)	0.3 (0.3–0.4)	0.4 (0.3–0.5)

	1990	2019	1990	2019	1990	2019	1990	2019	1990	2019	1990	2019
	Females	Females	Females	Females	Females	Females	Females	Females	Females	Females	Females	Females
Angola	1.6 (0.8–3.5)	2.6 (1.4–4.4)	2.0 (1.3–3.0)	2.0 (1.3–2.9)	0.9 (0.6–1.3)	1.8 (1.3–2.4)	0.5 (0.3–0.7)	0.7 (0.5–0.9)	1.3 (0.8–1.9)	1.2 (0.9–1.7)	0.6 (0.4–0.9)	0.6 (0.4–1.0)
Botswana	3.3 (2.3–4.9)	5.9 (3.9–8.9)	2.8 (2.0–3.8)	3.7 (2.4–5.3)	2.0 (1.5–2.8)	5.0 (3.4–7.1)	0.6 (0.4–0.8)	1.0 (0.7–1.4)	1.2 (0.9–1.7)	1.3 (0.8–1.9)	0.1 (0.1–0.1)	0.1 (0.1–0.1)
Comoros	3.5 (1.9–5.5)	6.5 (3.9–9.8)	2.9 (1.6–3.9)	2.9 (1.8–4.0)	1.6 (0.9–2.2)	2.5 (1.8–3.3)	0.6 (0.4–0.9)	1.0 (0.8–1.2)	1.2 (0.7–1.7)	1.3 (0.9–1.7)	0.8 (0.4–1.1)	1.0 (0.7–1.3)
DRC	1.8 (1.0–3.6)	2.4 (1.5–4.0)	2.0 (1.5–2.9)	1.9 (1.3–2.8)	1.0 (0.8–1.4)	1.3 (0.9–1.7)	0.5 (0.4–0.6)	0.5 (0.3–0.8)	1.2 (0.8–1.7)	1.1 (0.6–1.6)	0.6 (0.4–1.0)	0.6 (0.4–0.9)
ESwatini	4.4 (2.4–8.8)	6.3 (3.5–10.9)	3.0 (2.2–4.0)	3.4 (2.1–5.0)	2.3 (1.7–3.1)	3.8 (2.4–5.4)	0.7 (0.5–0.9)	0.9 (0.6–1.3)	1.1 (0.8–1.6)	1.1 (0.7–1.6)	0.6 (0.4–0.8)	0.6 (0.3–0.9)
Lesotho	2.8 (1.9–4.2)	6.3 (3.5–11.9)	2.1 (1.6–2.7)	3.9 (2.5–5.7)	1.2 (0.9–1.7)	3.3 (2.1–4.9)	0.4 (0.2–0.5)	0.8 (0.5–1.1)	1.0 (0.6–1.4)	1.4 (0.8–2.1)	0.5 (0.3–0.7)	0.7 (0.4–1.0)
Madagascar	3.0 (1.9–4.5)	3.9 (2.4–6.0)	2.4 (1.4–3.0)	2.4 (1.4–3.4)	1.2 (0.9–1.5)	1.6 (1.0–2.3)	0.5 (0.4–0.7)	0.7 (0.5–1.0)	1.1 (0.8–1.4)	1.2 (0.8–1.6)	0.8 (0.6–1.0)	0.9 (0.7–1.3)
Malawi	2.7 (2.0–3.5)	4.0 (2.3–6.2)	1.6 (1.2–2.1)	1.4 (1.0–1.9)	1.2 (1.0–1.5)	1.9 (1.4–2.5)	1.4 (1.0–1.9)	1.8 (1.4–2.3)	0.8 (0.5–1.0)	0.6 (0.4–0.9)	1.0 (0.8–1.2)	1.0 (0.7–1.3)
Mauritius	3.2 (2.9–3.5)	5.2 (4.1–6.6)	4.9 (4.5–5.4)	3.5 (2.8–4.2)	2.5 (2.3–2.8)	3.3 (2.6–4.0)	0.6 (0.6–0.7)	0.9 (0.8–1.1)	1.8 (1.5–2.4)	1.4 (1.0–1.7)	0.6 (0.5–0.6)	0.4 (0.3–0.5)
Mozambique	3.2 (2.1–4.6)	4.8 (3.3–6.8)	2.5 (1.5–3.6)	2.8 (1.7–4.1)	1.1 (0.8–1.3)	2.3 (1.5–3.4)	0.4 (0.3–0.5)	0.7 (0.5–1.0)	1.2 (0.7–1.7)	1.4 (0.9–2.0)	0.9 (0.6–1.2)	1.1 (0.7–1.6)
Namibia	2.6 (1.9–3.6)	3.7 (2.6–5.2)	2.2 (1.6–3.0)	2.3 (1.6–3.2)	1.1 (0.9–1.4)	2.6 (1.9–3.6)	0.9 (0.7–1.1)	1.3 (0.9–1.8)	0.8 (0.6–1.1)	0.8 (0.5–1.0)	0.5 (0.4–0.7)	0.5 (0.3–0.7)
Seychelles	5.5 (4.5–7.9)	8.9 (7.0–10.8)	2.9 (2.4–3.7)	3.0 (2.4–3.8)	2.5 (2.2–2.9)	3.8 (3.2–4.5)	0.8 (0.7–0.9)	1.0 (0.8–1.2)	1.5 (1.1–1.8)	1.3 (1.0–1.7)	0.4 (0.3–0.5)	0.4 (0.3–0.5)
South Africa	3.6 (2.9–4.2)	4.7 (3.6–5.7)	1.8 (1.4–2.2)	2.3 (1.6–2.7)	2.7 (2.2–3.4)	3.6 (3.1–4.1)	0.8 (0.7–0.9)	0.8 (0.7–0.9)	0.9 (0.7–1.1)	0.9 (0.6–1.0)	0.5 (0.4–0.6)	0.4 (0.4–0.5)
Tanzania	3.7 (2.3–5.3)	5.3 (4.1–6.6)	2.7 (1.6–3.5)	2.8 (1.8–3.6)	1.5 (1.2–1.8)	2.4 (1.9–2.9)	0.6 (0.5–0.8)	1.1 (0.9–1.4)	1.3 (1.0–1.7)	1.3 (1.0–1.8)	0.9 (0.7–1.1)	1.1 (0.8–1.4)
Zambia	4.2 (2.5–6.5)	5.8 (4.2–7.7)	3.5 (2.1–4.4)	3.1 (1.9–4.3)	2.0 (1.4–2.6)	3.0 (2.0–4.4)	0.8 (0.5–1.2)	1.2 (0.8–1.7)	1.5 (1.0–2.1)	1.4 (1.0–1.9)	1.0 (0.7–1.4)	1.1 (0.8–1.4)
Zimbabwe	4.5 (3.5–5.8)	7.5 (5.2–10.1)	3.7 (2.8–4.6)	5.5 (3.3–7.5)	3.5 (3.0–4.2)	6.4 (4.6–8.4)	0.5 (0.4–0.6)	0.7 (0.5–1.0)	1.7 (1.3–2.1)	1.7 (1.2–2.4)	1.1 (0.8–1.4)	1.4 (0.9–1.9)
SADC	3.0 (2.3–4.1)	4.2 (3.4–5.2)	2.3 (1.7–2.7)	2.5 (1.7–3.1)	1.7 (1.5–1.9)	2.4 (2.0–2.9)	0.6 (0.6–0.7)	0.8 (0.7–1.0)	1.2 (1.0–1.4)	1.2 (0.9–1.5)	0.7 (0.6–0.9)	0.8 (0.6–1.0)

*Note*: Cancer‐type, age‐standardized mortality and 95% uncertainty intervals (UIs) were calculated for each country using the Cause of Death Ensemble model 22 (Foremans, 2012), Vander Hoorn S, 2004).

Abbreviations: ‐, Not applicable for sex; BMI, body, mass index (kg/m^2^); DRC, Democratic, Republic of the Congo.

Mortality rate analysis of kidney cancers for the SADC in 2019 shows the rate to be 1.6 (1.3–2.1) for males and 0.8 (0.7–1.0) for females. Gallbladder/biliary tract cancer mortality remained constant at 1.0 (0.8–1.2) in both 1990 and 2019 for males. It also remained constant in females at 1.2 (1.0–1.4) for both periods. Thyroid cancer mortality increased slightly from 0.3 (0.3–0.4) in 1990 to 0.4 (0.3–0.5) in 2019 among males, and 0.7 (0.6–0.9) to 0.8 (0.6–1.0) among females.

### 3.3 Age standardized annualized rate of change for mortality

Table 4 shows age‐standardized prevalence per 100,000 population for five leading high‐BMI‐related cancers (breast, colon/rectal, oesophageal, prostate, and liver) for the period 1990 and 2019.

**TABLE 4:** Age‐standardized prevalence rate per 100,000 population due to 5 leading obesity‐related cancers in Southern Africa development community countries, 1990 and 2019.

	Breast		Esophageal		Colon and rectal		Liver		Leukemia	
	1990	2019	1990	2019	1990	2019	1990	2019	1990	2019
	Males	Males	Males	Males	Males	Males	Males	Males	Males	Males
Angola	3.2 (2.2–4.5)	3.9 (2.7–5.4)	19.4 (8.4–27.5)	15.3 (8.4–20.8)	22.6 (15.3–32.3)	32.6 (24.6–41.4)	3.1 (2.3–4.3)	3.3 (2.5–4.2)	5.2 (3.3–7.4)	4.5 (3.2–6.0)
Botswana	6.7 (4.9–9.0)	9.8 (7.0–13.4)	27.9 (15.6–40.1)	28.5 (18.0–38.2)	36.8 (28.3–47.3)	76.7 (56.3–97.1)	1.1 (0.4–3.7)	2.3 (1.6–3.1)	4.7 (3.2–6.7)	5.1 (3.3–6.9)
Comoros	9.5 (5.2–14.4)	10.1 (5.9–16.1)	21.6 (9.7–31.6)	16.9 (11.0–25.5)	20.7 (11.9–27.9)	25.9 (18.2–36.1)	3.4 (1.4–7.7)	3.1 (1.8–6.1)	3.8 (2.0–5.3)	3.8 (2.4–5.5)
DRC	3.8 (2.4–7.2)	3.8 (2.4–7.5)	17.4 (7.9–24.7)	13.9 (7.5–20.7)	19.2 (14.5–31.1)	19.5 (12.8–36.4)	2.9 (2.3–3.6)	2.5 (1.8–3.5)	4.6 (3.5–6.1)	3.9 (2.6–5.6)
ESwatini	6.3 (4.3–8.5)	8.1 (4.9–12.9)	34.4 (18.9–47.4)	37.8 (23.8–51.5)	33.8 (24.9–45.7)	54.2 (34.6–76.0)	7.4 (3.9–19.4)	40.6 (9.2–75.5)	4.9 (3.4–6.0)	5.7 (3.6–7.6)
Lesotho	5.4 (3.9–7.4)	7.0 (4.6–10.2)	24.9 (13.6–34.1)	31.4 (20.1–40.8)	20.8 (15.9–30.1)	38.4 (28.2–52.0)	6.7 (3.4–19.5)	27.9 (9.3–46.7)	4.3 (3.2–5.5)	6.1 (4.1–8.0)
Madagascar	9.5 (6.4–13.5)	8.7 (5.5–13.5)	17.1 (8.8–25.1)	13.3 (8.4–20.4)	18.9 (15.1–23.0)	20.8 (14.4–29.0)	3.1 (1.5–7.3)	2.6 (1.3–5.0)	3.7 (2.9–4.6)	3.1 (2.1–4.4)
Malawi	12.2 (8.7–16.8)	13.4 (9.3–18.6)	33.8 (26.3–44.4)	46.1 (33.3–62.8)	13.5 (11.2–16.1)	20.7 (15.2–26.8)	3.8 (2.5–6.5)	3.9 (3.0–5.0)	3.8 (2.9–4.7)	3.4 (2.3–4.6)
Mauritius	0.6 (0.5–0.7)	1.5 (1.2–2.0)	5.6 (5.1–6.1)	5.4 (4.3–7.0)	42.4 (38.8–46.6)	102.2 (81.9–126.7)	2.3 (2.1–2.5)	2.8 (2.0–3.8)	4.2 (3.9–4.5)	3.8 (3.1–4.6)
Mozambique	1.0 (0.7–1.3)	1.2 (0.9–1.5)	12.6 (9.5–15.9)	18.1 (13.4–24.5)	12.3 (10.3–14.8)	26.6 (19.2–34.8)	2.3 (1.3–5.0)	5.7 (3.6–7.8)	4.7 (3.6–6.0)	5.4 (4.0–7.1)
Namibia	7.4 (5.3–10.2)	12.4 (9.0–16.6)	5.0 (3.6–6.7)	6.5 (4.8–8.5)	16.9 (12.9–21.1)	35.2 (28.1–44.4)	2.1 (1.0–5.2)	5.0 (3.7–6.6)	2.8 (2.1–3.6)	3.1 (2.3–3.9)
Seychelles	1.0 (0.8–1.3)	2.4 (1.8–3.1)	14.2 (11.5–16.7)	14.5 (11.9–17.6)	71.6 (61.0–81.9)	165.4 (140.1–198.1)	11.9 (9.8–14.5)	9.0 (7.1–11.1)	8.4 (6.9–10.2)	7.7 (6.0–9.3)
South Africa	4.5 (3.8–5.6)	4.9 (4.3–5.8)	28.2 (19.5–37.8)	21.0 (18.2–27.4)	38.8 (33.0–49.2)	52.8 (46.6–61.0)	7.7 (4.3–16.4)	9.1 (7.6–10.6)	4.5 (3.5–5.3)	4.3 (3.0–5.0)
Tanzania	13.5 (9.4–19.3)	13.5 (8.7–20.3)	23.4 (13.4–34.8)	19.0 (11.7–30.3)	23.0 (18.4–30.0)	31.2 (23.4–44.2)	2.3 (1.8–3.0)	2.7 (1.9–3.7)	4.4 (3.5–5.7)	4.4 (2.9–6.3)
Zambia	13.1 (9.3–18.6)	15.1 (9.7–23.0)	26.3 (14.7–33.3)	24.2 (16.1–34.3)	27.0 (21.3–33.6)	45.0 (32.8–57.9)	2.5 (1.6–4.3)	3.6 (2.8–4.6)	4.7 (3.5–6.2)	4.5 (3.3–5.7)
Zimbabwe	2.4 (1.9–3.0)	2.7 (2.0–3.5)	29.6 (24.4–35.7)	30.3 (23.4–39.2)	31.3 (26.7–36.3)	37.3 (28.9–46.1)	15.2 (10.4–28.0)	15.9 (10.7–25.9)	3.8 (3.0–4.7)	3.4 (2.5–5.2)

SADC	6.4 (5.3–7.9)	6.9 (5.4–9.0)	22.5 (14.6–28.6)	19.4 (14.4–24.8)	24.8 (21.5–29.5)	31.7 (26.7–38.5)	4.6 (3.2–8.1)	4.9 (4.3–5.6)	4.4 (3.6–5.3)	4.2 (3.1–5.2)

	1990	2019	1990	2019	1990	2019	1990	2019	1990	2019
	Females	Females	Females	Females	Females	Females	Females	Females	Females	Females
Angola	104.9 (80.8–131.2)	171.6 (129.8–222.7)	10.2 (4.7–14.8)	7.4 (3.6–10.7)	16.6 (10.7–26.1)	23.7 (17.8–31.3)	2.0 (1.3–2.8)	1.7 (1.3–2.3)	3.2 (1.9–4.9)	2.4 (1.6–3.3)
Botswana	181.0 (137.6–236.5)	356.8 (240.8–510.8)	11.7 (5.7–17.2)	10.3 (5.2–15.1)	27.0 (19.6–37.3)	53.0 (35.4–75.7)	0.9 (0.5–1.3)	1.0 (0.6–1.4)	2.8 (2.0–3.8)	3.6 (2.3–5.2)
Comoros	109.3 (65.1–148.8)	176.6 (134.1–228.3)	16.9 (8.6–23.9)	14.1 (8.8–19.6)	18.7 (10.8–27.2)	25.5 (18.3–33.6)	2.8 (1.6–4.1)	2.6 (1.9–3.5)	2.5 (1.4–3.5)	2.5 (1.8–3.4)
DRC	133.5 (108.9–162.9)	181.2 (136.2–234.6)	10.2 (4.3–14.5)	8.4 (4.0–13.2)	15.2 (11.9–19.6)	15.7 (10.5–23.8)	2.1 (1.6–2.7)	2.0 (1.4–2.7)	2.7 (1.9–3.7)	2.0 (1.2–3.1)
ESwatini	142.6 (109.5–176.5)	212.5 (140.2–305.5)	11.2 (5.4–16.2)	8.8 (4.7–13.3)	22.7 (17.5–29.5)	30.8 (19.8–44.7)	4.3 (2.9–6.2)	4.3 (2.5–7.4)	2.9 (2.2–3.6)	3.1 (2.0–4.4)
Lesotho	114.0 (89.8–145.4)	226.3 (150.0–330.7)	9.0 (4.7–12.1)	11.1 (5.7–16.3)	14.3 (10.8–19.8)	26.3 (16.9–37.0)	3.9 (2.7–6.4)	5.2 (3.0–8.7)	2.1 (1.4–2.8)	3.5 (2.2–5.0)
Madagascar	115.5 (97.0–136.6)	147.5 (111.7–192.7)	15.5 (8.2–19.8)	13.3 (7.9–19.3)	17.5 (13.0–22.3)	20.3 (14.7–28.1)	2.8 (2.1–3.7)	2.4 (1.7–3.3)	2.6 (1.9–3.5)	2.2 (1.6–2.8)
Malawi	105.8 (85.9–126.9)	141.7 (108.0–178.4)	25.8 (20.3–32.8)	23.6 (17.0–32.6)	11.2 (9.1–13.5)	14.8 (11.1–19.2)	3.0 (2.3–3.7)	2.0 (1.5–2.6)	2.4 (1.8–3.0)	1.9 (1.3–2.7)
Mauritius	198.5 (178.7–220.9)	465.9 (379.6–568.0)	3.6 (3.3–3.9)	2.7 (2.1–3.3)	36.6 (33.4–40.0)	76.0 (61.4–92.7)	1.4 (1.3–1.6)	1.5 (1.2–1.8)	3.4 (3.2–3.7)	3.0 (2.5–3.7)
Mozambique	104.2 (84.9–125.2)	166.1 (119.0–224.2)	3.7 (2.6–4.8)	3.8 (2.6–5.4)	11.8 (9.1–14.5)	19.6 (14.0–26.8)	1.9 (1.4–2.5)	2.1 (1.5–3.0)	3.6 (2.6–5.0)	3.5 (2.4–4.8)
Namibia	147.5 (115.1–177.9)	343.8 (244.4–489.3)	1.8 (1.3–2.4)	1.6 (1.1–2.2)	14.8 (11.1–18.9)	26.1 (18.9–35.5)	1.6 (1.2–2.2)	2.1 (1.5–2.8)	2.0 (1.5–2.6)	2.1 (1.5–2.9)
Seychelles	220.3 (194.8–250.6)	472.9 (399.2–551.5)	2.9 (2.4–3.4)	2.9 (2.3–3.7)	59.7 (53.0–67.1)	124.6 (103.6–147.6)	4.5 (3.8–5.4)	3.0 (2.4–3.7)	6.2 (5.3–7.1)	5.1 (4.2–6.4)
South Africa	198.8 (176.9–223.2)	241.6 (212.9–272.6)	12.2 (8.4–15.0)	8.0 (6.8–9.9)	29.5 (25.0–34.9)	35.2 (30.5–40.9)	4.7 (3.5–6.1)	3.3 (2.7–4.2)	3.2 (2.6–3.6)	2.6 (2.1–3.1)
Tanzania	101.8 (84.7–119.7)	155.8 (124.8–188.6)	16.7 (8.9–22.1)	13.8 (8.4–19.5)	19.4 (15.7–23.3)	28.1 (22.6–34.6)	1.9 (1.5–2.3)	1.9 (1.5–2.4)	2.8 (2.1–3.6)	2.9 (2.2–3.8)
Zambia	131.4 (109.0–160.3)	183.3 (139.1–237.0)	20.4 (11.4–27.5)	15.1 (9.0–21.7)	25.1 (16.7–35.3)	35.2 (25.1–48.5)	2.1 (1.6–2.8)	2.0 (1.5–2.6)	3.5 (2.3–4.9)	2.9 (2.1–3.7)
Zimbabwe	174.0 (146.5–203.3)	249.3 (182.8–330.7)	10.6 (8.4–12.9)	13.9 (9.2–19.2)	29.0 (24.6–34.4)	38.5 (28.2–50.7)	8.9 (7.1–10.9)	12.3 (8.5–17.7)	2.5 (2.0–3.1)	2.5 (1.8–3.4)
SADC	139.2 (125.2–154.5)	186.8 (156.2–222.5)	12.7 (8.0–15.2)	10.5 (7.0–13.6)	20.3 (17.8–23.1)	24.9 (21.0–29.5)	3.1 (2.7–3.6)	2.7 (2.2–3.2)	2.9 (2.4–3.5)	2.5 (2.0–3.2)

*Note*: Cancer‐type, age‐standardized prevalence rates and 95% uncertainty intervals (UIs) were calculated for each country using the Cause of Death Ensemble model 22 (Foremans, 2012), Vander Hoorn S, 2004).

Abbreviations: BMI, body mass index (kg/m^2^); DRC, Democratic Republic of the Congo.

#### 3.3.1 Breast cancer

For all 16 countries combined, the most prevalent cancer was breast cancer in females with an estimated prevalence of 139.2 (125.2–154.5) in 1990 increasing substantially to 186.8 (156.2–222.5) in 2019. The 2019 prevalence was 1.34 (1.25–1.44) times that for 1990. Seychelles, 472.9 (399.2–551.5) and Mauritius, 465.9 (379.6–568.0) had the highest prevalence of female breast cancer in 2019, each posting a 2.5‐fold the SADC prevalence. In 2019 the bottom prevalence of breast cancer was in Malawi, 141.7 (108.0–178.4), and Madagascar, 147.5 (111.7–192.7). The 2019 breast cancer prevalence for males increased in all countries relative to 1990 with the top prevalence in Zambia, 15.1 (9.7–23.0), Tanzania, 13.5 (8.7–20.3), Malawi, 13.4 (9.3–18.6), and Namibia, 12.4 (9.0–16.6).

#### 3.3.2 Colon and rectal cancer

Overall, colon/rectal cancer among males increased 1.28‐fold from 24.8 (21.5–29.5) in 1990 to 31.7 (26.7–38.5) in 2019, while in females, they increased by 1.23‐fold from 20.3 (17.8–23.1) to 24.9 (21.0–29.5). Seychelles and Mauritius posted disproportionately high prevalence for colon/rectal cancer for both sexes during the same period. The prevalence for colon/rectal cancer were 165.4 (140.1–198.1) for males and 124.6 (103.6–147.6) for females for Seychelles while that of Mauritius was 102.2 (81.9–126.7) for males, and 198.5 (178.7–220.9) for females.

#### 3.3.3 Oesophageal cancer

The prevalence of oesophageal cancer for males decreased from 22.5 (14.6–28.6) in 1990 to 19.4 (14.4–24.8) in 2019, while for females there was a decrease from 12.7(8.0–15.2) to 10.5 (7.0–13.6).

Table 5 shows age‐standardized prevalence per 100,000 population for ovarian, uterine, pancreatic, kidney, gallbladder/biliary tract, and thyroid cancers for 1990 and 2019. The prevalence of uterine cancer in the 16 SADC countries combined increased from 12.7 (9.8–14.7) in 1990 to 17.5(12.8–21.7) in 2019. Prevalence by individual countries varied with unusually high prevalence observed for Mauritius 85.2 (66.7–106.1), Seychelles 71.3 (55.1–89.8), and Botswana 42.7 (26.1–64.9). Prevalence in all other countries ranged from 8.7 (5.9–11.9) in Malawi to 37.7 (20.3–53.3) in Zimbabwe in 2019. For ovarian cancer, the prevalence increased from 11.9 (9.0–17.0) in 1990 to 17.8 (14.0–22.3) in 2019. Seychelles 74.4 (57.9–2.4) had the highest prevalence followed by Mauritius 47.6 (36.7–60.5), Zimbabwe 31.8 (21.2–43.0) and Botswana 30.3 (18.4–46.4) respectively. For males, pancreatic cancer prevalence in Eswatini 11.7 (7.4–17.5), Seychelles 9.7 (8.2–11.5), and Botswana 8.7 (6.3–11.5) were 1.9 to 2.5‐times the regional rate of 4.6 (3.7–5.4). Among females, the regional rate in 2019 of 3.5 (3.0–4.2) was mostly driven by the higher prevalence observed in Zimbabwe 9.0 (6.6–11.9), and Botswana 7.3 (5.1–10.2) respectively.

**TABLE 5:** Age‐standardized prevalence rate per 100,000 population of other obesity‐related cancers in Southern Africa development community countries, 1990 and 2019.

	Uterine cancer		Ovarian		Pancreatic		Kidney		Gallbladder/biliary tract		Thyroid	
	1990	2019	1990	2019	1990	2019	1990	2019	1990	2019	1990	2019
	Males	Males	Males	Males	Males	Males	Males	Males	Males	Males	Males	Males
Angola	‐	‐	‐	‐	3.0 (2.0–4.4)	4.4 (3.3–5.6)	2.7 (1.7–4.7)	5.0 (3.1–7.4)	1.1 (0.8–1.5)	1.1 (0.8–1.6)	0.9 (0.6–1.4)	1.8 (1.3–2.5)
Botswana	‐	‐	‐	‐	4.7 (3.7–6.0)	8.7 (6.3–11.5)	3.3 (2.3–4.5)	10.8 (7.7–14.6)	1.0 (0.7–1.5)	1.2 (0.9–1.8)	0.7 (0.5–0.9)	1.8 (1.2–2.6)
Comoros	‐	‐	‐	‐	3.5 (2.1–4.5)	4.2 (2.8–5.7)	2.8 (1.3–4.0)	4.9 (3.0–7.5)	0.9 (0.5–1.2)	0.9 (0.6–1.2)	1.3 (0.4–2.0)	3.0 (1.8–4.6)
DRC	‐	‐	‐	‐	2.9 (2.1–3.9)	3.0 (2.0–4.1)	2.5 (1.8–3.4)	2.8 (1.5–4.5)	1.0 (0.7–1.3)	0.9 (0.5–1.2)	0.9 (0.7–1.4)	1.2 (0.8–1.8)
ESwatini	‐	‐	‐	‐	6.6 (4.4–9.8)	11.7 (7.4–17.5)	4.5 (2.8–6.7)	11.0 (6.5–17.5)	1.1 (0.8–1.4)	1.2 (0.9–1.7)	1.9 (1.3–2.6)	4.7 (2.8–6.8)
Lesotho	‐	‐	‐	‐	3.9 (2.7–5.4)	7.4 (5.4–10.3)	2.2 (1.3–3.3)	5.9 (3.8–8.4)	0.9 (0.6–1.1)	1.2 (0.8–1.6)	1.1 (0.8–1.6)	2.7 (1.7–3.9)
Madagascar	‐	‐	‐	‐	2.3 (1.7–3.0)	2.6 (1.6–3.8)	2.4 (1.6–3.6)	3.0 (1.8–4.7)	0.8 (0.5–1.0)	0.7 (0.5–1.0)	1.4 (1.1–1.9)	2.1 (1.3–3.1)
Malawi	‐	‐	‐	‐	2.3 (1.8–2.9)	3.4 (2.4–4.6)	6.1 (4.7–8.1)	9.5 (6.1–13.7)	0.7 (0.5–0.9)	0.8 (0.6–1.0)	1.2 (0.9–1.5)	2.1 (1.4–3.0)
Mauritius	‐	‐	‐	‐	5.1 (4.7–5.5)	6.5 (5.1–7.9)	9.2 (8.4–10.0)	20.9 (16.4–26.2)	1.8 (1.3–2.0)	1.2 (0.9–1.5)	5.5 (4.4–6.4)	8.5 (6.3–11.2)
Mozambique	‐	‐	‐	‐	2.3 (1.8–2.8)	4.4 (3.1–6.3)	1.6 (1.3–2.1)	4.1 (2.6–6.0)	0.9 (0.6–1.2)	1.4 (1.0–1.8)	1.1 (0.7–1.5)	4.1 (2.8–5.8)
Namibia	‐	‐	‐	‐	2.2 (1.7–2.8)	4.4 (3.4–5.5)	3.0 (2.1–4.0)	9.0 (6.3–12.2)	0.6 (0.5–0.8)	0.7 (0.6–1.0)	1.2 (0.8–1.7)	4.4 (2.9–6.5)
Seychelles	‐	‐	‐	‐	7.7 (6.4–9.0)	9.7 (8.2–11.5)	10.6 (8.8–12.7)	26.0 (21.2–31.9)	1.8 (1.3–2.3)	1.7 (1.3–2.1)	4.0 (3.1–5.1)	7.0 (5.3–9.0)
South Africa	‐	‐	‐	‐	5.6 (4.6–7.4)	7.4 (6.5–8.5)	5.3 (4.7–6.3)	8.6 (7.5–9.7)	0.8 (0.6–1.0)	0.8 (0.7–1.0)	2.4 (2.1–2.9)	3.2 (2.7–3.9)
Tanzania	‐	‐	‐	‐	3.4 (2.6–4.3)	4.3 (3.2–6.0)	3.3 (2.5–4.3)	6.4 (4.2–11.0)	1.0 (0.7–1.4)	1.0 (0.7–1.5)	1.9 (1.3–2.7)	3.8 (2.5–5.9)
Zambia	‐	‐	‐	‐	4.2 (3.4–5.0)	5.8 (4.2–8.0)	3.8 (2.0–5.3)	7.9 (4.4–12.0)	1.0 (0.8–1.3)	1.2(0.9–1.5)	1.8 (1.3–2.3)	5.3 (3.6–7.3)
Zimbabwe	‐	‐	‐	‐	4.8 (4.1–5.5)	6.5 (4.9–8.1)	2.7 (2.2–3.2)	3.8 (2.7–5.1)	1.5 (1.2–1.8)	1.2 (0.9–1.8)	2.8 (2.1–3.6)	3.7 (2.6–5.2)
SADC					3.7 (3.1–4.3)	4.6 (3.7–5.4)	3.5 (3.1–4.2)	5.5 (4.3–7.0)	0.9 (0.8–1.1)	1.0 (0.8–1.2)	1.6 (1.4–1.9)	2.7 (2.1–3.5)

	1990	2019	1990	2019	1990	2019	1990	1990	2019	1990	2019	1990
	Females	Females	Females	Females	Females	Females	Females	Females	Females	Females	Females	Females
Angola	8.1 (5.2–12.2)	13.1 (8.5–19.9)	5.4 (2.8–12.8)	10. (6.0–18.1)	1.4 (1.0–1.9)	2.6 (2.0–3.4)	1.4 (0.8–2.9)	2.9 (2.0–4.1)	1.2 (0.8–1.9)	1.2 (0.9–1.7)	3.4 (2.1–5.0)	6.0 (3.6–9.7)
Botswana	17.6 (12.0–25.8)	42.7 (26.1–64.9)	13.7 (8.9–20.1)	30.3 (18.4–46.4)	3.1 (2.2–4.1)	7.3 (5.1–10.2)	2.2 (1.5–3.0)	6.6 (4.3–9.7)	1.2 (0.9–1.7)	1.3 (0.9–2.0)	1.1 (0.7–1.6)	2.5 (1.5–3.7)
Comoros	11.6 (5.4–16.9)	19.1 (12.0–27.0)	12.3 (5.1–19.9)	27.5 (15.4–42.7)	2.4 (1.4–3.2)	3.6 (2.7–4.7)	1.7 (0.8–2.8)	4.0 (2.7–5.3)	1.2 (0.7–1.7)	1.2 (0.8–1.7)	7.1 (2.0–11.6)	16.4 (9.6–25.7)
DRC	9.0 (6.4–12.8)	11.0 (7.3–16.6)	6.4 (3.6–13.3)	9.3 (5.8–15.4)	1.6 (1.2–2.0)	1.9 (1.4–2.6)	1.5 (1.0–2.2)	1.91((0.1–2.6)	1.2(0.8–1.7)	1.1 (0.6–1.6)	3.8 (2.5–5.5)	5.1 (3.2–7.7)
ESwatini	15.6 (11.4–21.5)	22.3 (12.5–35.1)	16.8 (9.1–34.2)	26.6 (13.8–46.2)	3.5 (2.6–4.8)	5.9 (3.8–8.2)	2.3 (1.7–2.9)	4.0 (2.5–5.9)	1.1 (0.8–1.5)	1.1 (0.6–1.6)	5.5 (3.5–8.0)	7.3 (3.9–12.1)
Lesotho	9.8 (7.2–13.6)	21.8 (12.7–33.7)	10.3 (6.6–15.4)	23.6 (12.8–45.5)	1.9 (1.4–2.6)	5.1 (3.3–7.3)	1.4 (0.8–1.9)	3.1 (2.0–4.6)	0.9 (0.6–1.3)	1.3 (0.8–2.0)	3.9 (2.5–5.6)	7.2 (3.6–11.7)
Madagascar	11.1 (6.7–14.0)	14.0 (8.4–20.2)	11.5 (7.1–19.2)	16.2 (9.9–25.3)	1.7 (1.3–2.1)	2.3 (1.5–3.2)	1.7 (1.2–2.8)	2.6 (1.9–3.4)	1.1 (0.8–1.4)	1.1 (0.8–1.6)	9.8 (6.8–13.9)	13.3 (9.0–19.0)
Malawi	7.5 (5.4–9.6)	8.7 (5.9–11.9)	10.0 (7.3–13.2)	16.8 (9.2–26.5)	1.8 (1.4–2.2)	2.9 (2.1–3.7)	4.2 (2.8–7.1)	7.0 (4.9–9.4)	0.7 (0.5–0.9)	0.6 (0.4–0.8)	11.4 (8.1–15.8)	15.4 (9.1–24.9)
Mauritius	86.5 (76.9–96.8)	85.2 (66.7–106.1)	22.3 (20.0–24.9)	47.6 (36.7–60.5)	3.6 (3.3–3.9)	4.4 (3.5–5.4)	8.3 (7.0–9.7)	16.1 (12.1–20.5)	2.0 (1.8–2.7)	1.8 (1.4–2.3)	14.9 (12.7–17.2)	22.0 (16.4–28.1)
Mozambique	8.8 (5.3–12.7)	15.5 (9.1–24.0)	10.9 (6.9–16.5)	19.3 (12.6–27.6)	1.6 (1.3–2.0)	3.5 (2.3–5.0)	1.2 (0.9–1.8)	2.9 (1.9–4.1)	1.1 (0.7–1.6)	1.3 (0.8–2.0)	6.6 (4.6–9.6)	14.8 (8.6–23.6)
Namibia	11.6 (8.2–15.7)	22.3 (14.3–32.6)	9.7 (6.5–13.7)	17.1 (11.3–25.1)	1.6 (1.3–2.0)	3.9 (2.8–5.3)	2.4 (1.8–3.2)	6.0 (4.0–8.7)	0.8 (0.5–1.1)	0.8 (0.5–1.1)	5.6 (3.5–8.0)	11.4 (7.0–18.3)
Seychelles	46.8 (37.2–58.6)	71.3 (55.1–89.8)	37.1 (30.7–51.1)	74.4 (57.9–92.4)	3.6 (3.1–4.2)	5.5 (4.6–6.5)	7.4 (6.0–9.0)	12.3 (9.8–15.4)	1.7 (1.2–2.0)	1.5 (1.2–2.0)	7.9 (6.3–9.9)	12.9 (9.9–16.7)
South Africa	14.2 (11.8–17.0)	22.4 (16.6–26.9)	16.0 (13.7–19.1)	21.4 (16.5–27.3)	4.1 (3.3–5.1)	5.4 (4.8–6.2)	3.9 (3.4–4.5)	4.8 (4.2–5.6)	0.9 (0.7–1.2)	0.9 (0.7–1.1)	8.3 (6.9–9.6)	7.9 (6.4–10.8)
Tanzania	13.2 (8.1–17.7)	19.9 (12.9–26.3)	13.9 (8.4–20.2)	22.9 (16.7–29.8)	2.2 (1.8–2.6)	3.5 (2.8–4.1)	2.1 (1.5–2.9)	4.9 (3.8–6.6)	1.2 (0.9–1.6)	1.3 (1.0–1.8)	9.6 (6.6–13.4)	17.8 (12.1–26.2)
Zambia	15.3 (9.3–20.4)	24.0 (15.0–34.1)	15.1 (8.8–24.4)	26.1 (18.5–35.6)	2.9 (2.1–3.8)	4.4 (3.0–6.3)	2.3 (1.5–4.1)	5.1 (3.5–7.2)	1.5 (1.0–2.1)	1.4 (1.0–2.0)	10.8 (6.7–16.6)	21.5 (13.9–31.9)
Zimbabwe	26.1(18.9–32.9)	37.6 (20.3–53.3)	18.4 (14.1–24.1)	31.8 (21.2–43.0)	5.1 (4.3–6.1)	9.0 (6.6–11.9)	1.9 (1.5–2.4)	2.9 (2.0–4.0)	1.7 (1.4–2.2)	1.7 (1.2–2.4)	15.4 (9.9–20.6)	21.0 (10.6–31.1)
SADC	12.7 (9.8–14.7)	17.5 (12.8–21.7)	11.9 (9.0–17.0)	17.8 (14.0–22.3)	2.5 (2.2–2.9)	3.5 (3.0–4.2)	2.4 (2.0–3.1)	3.7 (3.1–4.5)	1.2 (1.0–1.4)	1.1 (0.9–1.5)	7.8 (6.4–9.2)	11.4 (8.3–15.1)

*Note*: Cancer‐type, age‐standardised prevalence rates and 95% uncertainty intervals (UIs) were calculated for each country using the Cause of Death Ensemble model 22 (Foremans, 2012), Vander Hoorn S, 2004).

Abbreviations: ‐, Not applicable for sex; BMI, Body mass index (kg/m^2^); DRC, Democratic Republic of the Congo.

## 4 DISCUSSION

This study was heavily guided and inspired by the serious lack of information on trends in morbidity and mortality associated with high‐BMI‐related cancers in SADC countries. It was also inspired by our report of 2021.^48^ To our knowledge, this study represents the most comprehensive study so far that has reported the estimates of the burden of cancer due to obesity in SADC countries. Escalation of such diseases and other NCDs in the SADC countries requires information to highlight the impact and changes of high‐BMI‐related cancer burden on the populations for each of the countries to inform strategies and guide interventions to reduce the burden. The generally negative AROCs in males and females for oesophageal and leukaemia cancers provides evidence that mortality for these cancers is trending in the right direction but at a pace not rapid enough to meet the WHO UN SDG Target 3.4.

The increasing burden of high‐BMI‐related or lifestyle‐related cancers in SADC between 1990 and 2019 could be explained by the epidemiological transition where populations move from contracting primarily communicable diseases to developing non‐communicable diseases due to concomitant changes in lifestyle, dietary, and environmental exposures as well as increased access to health care, a combination which can similarly shift the distribution of non‐communicable diseases or conditions characterized as “degenerative and man‐made diseases”.^55^, ^56^, ^57^ The other reason, in part, is the aging populations. The demographic transition theory suggests that as populations live longer and age substantially, there seems to be a correlated increase to lifetime risks for cancer. While the demographic transition proposes that neoplasms occur mostly in middle and old age, it is now known that they may not primarily or necessarily be caused by age‐related biological processes of ‘degeneration’ but also by exogenous factors including poor diet, limited physical activity, sedentary lifestyle, and excessive alcohol consumption, all of which are mediated by rapid urbanization in developing countries.^58^ The lifetime probability of developing cancer and cancer‐related mortality in developed countries has already reached 56.9% and 27.6% in men, and 51.9% and 21.7% in women respectively.^59^ African countries are at different stages of the demographic transitions, and even in‐country transitions are at different levels that is, transitions are not monolithic, therefore interventions should be targeted to specific demographic patterns for each country and within country by region, such as rural/urban and other country‐relevant subnational demarcations.

A major risk factor in developing cancer is obesity and/or high BMI. Generally, obesity is rising in developing countries; and the SADC is no exception. Among the many known and unknown reasons for this rise includes unhealthy diets and lack of preventive practices at both personal and policy levels^60^ combined with rising disposable income.^61^ Observed obesity increase in Africa is largely caused by increasing urbanization,^62^ high changes in diet composition such as readily available and relatively cheaper fast foods confounded by dialogs comparing healthier foods as being harder to access and costly^63^ reduced physical exercise, and sedentary life styles.^49^ SADC countries should, as a matter of urgency start funding and developing public health and behavioral research interventions targeting these unhealthy behaviors with a focus on diet quality interrogations, caloric intake and physical activity, and the effect of rapid urbanization on childhood obesity and thereby suggest solutions as a way forward.^64^

Apart from putting functional policies in place to effectively halt and reverse the occurrence of high‐BMI‐related cancers and mortality, African governments, especially in the SADC require investments in prevention and early detection and cancer care services. Currently, although the demand for cancer care has risen sharply, cancer care services are still limited and therefore, access to such is extremely difficult for many low‐income cancer patients. For example, overweight and obesity were strongly associated with the later stages of breast cancer at diagnosis (stage III, IV) in Egypt,^65^ however and perhaps due to lack of screening awareness among women with obesity, breast cancers were discovered quite late and when irreversible. Breast cancer survival for most cases is driven by early diagnosis. However, cancer survival rates are worse in the African population than in developed countries, with the 5‐year survival rate of women with breast cancer in Europe being 82%, while it ranges between 12% and 46% in parts of Africa.^66^ The call and need for SADC countries to invest in widespread cancer screening and early diagnosis and treatment together with preventive efforts at primary care facilities cannot be overstated.

It is our view that curtailing marketing of unhealthy food and promoting healthy ones, strengthening health promotion behaviors and awareness of risk factors may be valuable in contributing to the reduction of obesity and subsequently subduing the visible increases in high‐BMI‐related cancers. Health promotion messaging in SADC should target kidney, colon/rectal, pancreatic and breast cancers and encourage clinically tested methods of reducing BMI such as increasing personal levels of physical activity that produce results and adoption of dietary regimes that are equally effective. Several weight loss strategies have been successful, including dietary approaches such as low‐calorie intake combining with behavioral therapy and behavioral dietary changes to support long‐term weight loss maintenance.^67^, ^68^ Such strategies have been shown to reduce weight by approximately 3–5 kg after 1 year.^69^ It is also true that certain observational studies have shown that consistent weight loss reduces the risk of cancer and therefore investment in such not‐so‐expensive therapies may suffice for the SADC. However, such studies maybe confounded by the fact that people who lose weight or avoid weight gain may differ in other ways from people who do not. For example, people who have lower weight gain during adulthood have lower risks of colon/rectal cancer, kidney cancer, and ‐for postmenopausal women—breast, endometrial, and ovarian cancers,^70^, ^71^, ^72^ but counterfactually, individuals who have lower weight gain might also be more active or eat healthier.

The consumption of processed foods in Africa has increased in the last decades, with the downside impact of increased obesity^73^ and cancer. SADC regions may require stringent and enforceable regulations to reduce uncontrolled access to ultra‐processed foods.^73^ The influence of ultra‐processed foods (both imported and locally made) and poor food legislature, in addition to regulation enforcement may be notable factors driving the burden of obesity‐related cancers. In developed countries, an increase in colorectal cancers have been attributed, in part, to high consumption of ultra‐processed foods.^74^ The consumption of processed foods has increased in SADC countries. In addition to locally processed foods, substantial increases in importation of poorly regulated processed drinks and foods from developed countries into the SADC region have dramatically increased. This is likely driven by a combination of the public need for cheaper food alternatives, and the need for increased business opportunities for regional and local importers, who create much needed employment and income opportunities.^75^ Local food quality control has also been found lacking in SADC countries. Despite the well documented carcinogenic and health risk properties of mycotoxins, secondary fungal metabolites known to contaminate major staple foods in these regions, legislation controlling mycotoxin contamination of food is still limited in a number of SADC countries.^76^ In addition, SADC populations, young and old, will benefit from awareness campaigns on food quality and conscious consumption through food label evaluation, specifically for those foods that may be associated with obesity and cancer.

Specific HIV treatment regiments have been associated with Obesity.^77^ Considering that sub‐Saharan Africa has the highest burden of people living with HIV and who are on anti‐retroviral treatment, globally,^78^ it is plausible that HIV treatment associated increases in BMI could also be driving increases in BMI‐associated cancers in SADC. In addition, significantly improved survival of people living with HIV has resulted in notable increases in aging populations who may be at high risk of developing non‐AIDS cancer,^79^ and generally, increased risk of developing cancer due to advancing age.^80^

Other more costly medical interventions that may allow the SADC to counter cancer progression could involve last resort surgical cancer care services not ideal for national rollout such as bariatric surgery for weight loss. Although such services are extremely limited and beyond the poor majority who may be at risk of such cancers, again, the demand in Africa is rising, although this may be an effective cancer prevention strategy needed to reduce cancer‐related morbidity and mortality. Mortality studies have shown more robust protective hazards over 12.5 years for obesity related cancers due to such interventions as bariatric surgery, (HR = 0.62; CI: 0.49–0.78).^81^ On account of such overwhelming evidence, it is incumbent upon SADC countries to invest in surgical cancer care services as well. However, location‐specific and culturally appropriate non‐surgical interventions and strategies for weight loss are the most important since most SADC countries are generally poor and may not afford quick investment in surgical or indeed complex medical procedures.

This study reinforces conclusions of the IHME's Total Cancers report^47^ and the GLOBOCAN 2020 analysis of 34 cancer types showing that high cancer mortality rates in Africa demand a holistic approach toward control and management such as increasing cancer awareness through health education, adoption of primary and secondary prevention methods through health promotion, mitigating risk factors, improving cancer infrastructure and robust screening, diagnosis, and timely treatment.^45^, ^82^ A large portion of the most rapidly rising cancers are avoidable by implementing public health. Such information is critical to help highlight the impact, changes and trends of high‐BMI‐related cancer burden on the populations for each of the countries to inform strategies and interventions to act to hopefully reduce the burden.

This study has some limitations. Firstly, period and cohort effects were not examined, which ideally would have allowed differentiation of the shift of cancer‐related mortality risk by time periods and birth cohorts for each country so as to better appreciate effects of epidemiological and demographic transitions. To adequately capture time trends in mortality for each age‐group adjusting for period effects, future analysis should use age‐period‐cohort models to analyze time trends in the burden of high‐BMI‐related cancers.^83^ Secondly, specific population and country characteristics were not available to adjust for in the analyses. Future studies should tease out the intersection of obesity, different types of cancers (especially for breast cancer in females, and leukaemia, oesophageal, colon/rectal cancer in both sexes), and risk factors in the environment such as industrial pollution, air and water pollution as well as climate change. Other limitations of GBD estimates have been documented elsewhere.^84^, ^85^ Finally, information on country‐specific policies were not available to address cancers, cancer‐related care and mitigation.

## 5 CONCLUSION

Age‐standardized death rates due to high‐BMI‐related cancers, especially kidney, colon/rectal, pancreatic and breast cancer in the SADC increased substantially in 2019. In particular, mortality due to breast cancer among males posted higher rates compared to cancers such as gallbladder/biliary tract, and thyroid cancers. Breast and other cancers were also significantly higher both at individual country level and across the SADC. However, it is important to note that while the situation requires maximum attention and intervention as well as investment in a number of programs including education, prevention and culturally sensitive aspects to curb cancers and related NCDs, SADC countries do actually have a “window” of opportunity to halt and reverse these negative health outcomes before they become endemic and out of control. Simple but comprehensive and broad implementation of known interventions at individual and community levels maybe starting points to fight off effects of both obesity and related cancers while looking forward to more intense policy shifts significant at individual country levels. Further and more focused research to identify tailored location‐specific and more efficient strategies for weight loss and control, clinical trials of what works and what does not in different settings, as well as health promotion and health education are needed. Public funding for these strategies should be encouraged.

## AUTHOR CONTRIBUTIONS

PNG conceptualized the study, had access to raw data, analyzed data, wrote the first draft of the manuscript, and interpreted the data, Clara M Gona, Suha Ballout, Chabila C Mapoma, Sowmya R Rao, contributed to the epidemiological, policy implications sections, and strengthened the intellectual content and recommendations of the study. Ali H. Mokdad supervised the development of the study, critiqued earlier drafts, and shaped the overall interpretation in relation to previous related studies. The authors read and approved the final manuscript. Please note: The publication is under the banner GBD 2019 SADC BMI. A full author list will be provided upon acceptance of the paper.

## CONFLICT OF INTEREST STATEMENT

All authors report no conflicts.
